# Berberine promotes the recruitment and activation of brown adipose tissue in mice and humans

**DOI:** 10.1038/s41419-019-1706-y

**Published:** 2019-06-13

**Authors:** Lingyan Wu, Mingfeng Xia, Yanan Duan, Lina Zhang, Haowen Jiang, Xiaobei Hu, Hongmei Yan, Yiqiu Zhang, Yushen Gu, Hongcheng Shi, Jia Li, Xin Gao, Jingya Li

**Affiliations:** 1 State Key Laboratory of Drug Research, Shanghai Institute of Materia Medica, Chinese Academy of Sciences, Shanghai, P. R. China; 2 University of Chinese Academy of Sciences, Beijing, P.R. China; 3 Department of Endocrinology and Metabolism, Zhongshan Hospital, Fudan University, Shanghai, P. R. China; 4 Fudan Institute for Metabolic Diseases, Shanghai, P. R. China; 5 Department of Nuclear Medicine, Zhongshan Hospital, Fudan University, Shanghai, P. R. China

## Abstract

Brown adipose tissue (BAT) dissipates metabolic energy and mediates non-shivering thermogenesis, thereby boosting energy expenditure. Increasing BAT mass and activity is expected to be a promising strategy for combating obesity; however, few medications effectively and safely recruit and activate BAT in humans. Berberine (BBR), a natural compound, is commonly used as a nonprescription drug to treat diarrhea. Here, we reported that 1-month BBR intervention increased BAT mass and activity, reduced body weight, and improved insulin sensitivity in mildly overweight patients with non-alcoholic fatty liver disease. Chronic BBR treatment promoted BAT development by stimulating the expression of brown adipogenic genes, enhanced BAT thermogenesis, and global energy expenditure in diet-induced obese mice and chow-fed lean mice, Consistently, BBR facilitated brown adipocyte differentiation in both mouse and human primary brown preadipocytes. We further found that BBR increased the transcription of PRDM16, a master regulator of brown/beige adipogenesis, by inducing the active DNA demethylation of PRDM16 promoter, which might be driven by the activation of AMPK and production of its downstream tricarboxylic acid cycle intermediate α-Ketoglutarate. Moreover, chronic BBR administration had no impact on the BAT thermogenesis in adipose-specific AMPKa1 and AMPKa2 knockout mice. In summary, we found that BBR intervention promoted recruitment and activation of BAT and AMPK–PRDM16 axis was indispensable for the pro-BAT and pro-energy expenditure properties of BBR. Our findings suggest that BBR may be a promising drug for obesity and related metabolic disorders in humans partially through activating BAT.

## Introduction

Brown adipose tissue (BAT) is specialized to combust energy by non-shivering thermogenesis mediated by mitochondrial uncoupling protein 1 (UCP1)^1^. Two types of thermogenic adipocytes exist: classical brown adipocytes that are abundant in BAT with high levels of UCP1, and beige/brite adipocytes that are abundant in white adipose tissue (WAT) with low levels of UCP1 under basal conditions. However, beige/brite adipocytes have potential to express comparable levels of UCP1 to brown adipocytes in response to stimuli such as cold temperatures or β3-adrenergic receptor agonists^2^. Several crucial transcriptional factors involved in brown and beige/brite adipocyte development have recently been identified^3^. Among them, PRD1-BF1-RIZ1 homologous domain-containing 16 (PRDM16) acts as a master regulator of brown/beige adipogenesis through its interactions with transcriptional factors such as PPARγ, C/EBPβ, PPARγ co-activator 1α (PGC-1α), zinc finger protein 516, and C-terminal-binding protein 1^4–6^.

It has been demonstrated that considerable functional BAT exists in human adults^7–9^. The activity of human BAT is negatively correlated with age, BMI, fat mass, and fasting blood glucose (FBG), and positively correlated with resting metabolic rate^8–10^. BAT functions to increase energy expenditure and improve glucose metabolism and insulin sensitivity in humans^11^. Thus, pharmacological strategies targeting BAT provide an appealing option for obesity therapy, as few drugs have been identified to effectively and safely recruit or activate BAT in humans.

Berberine (BBR), an active product isolated from the medicinal plant *Rhizoma Coptidis*, is widely used as an anti-diarrhea drug and also reported to exert antidiabetic and antihyperlipidemic effects in humans^12,13^. Our recent study showed that a 4-month BBR intervention significantly decreased body weight (BW) and liver fat content in patients with non-alcoholic fatty liver disease (NAFLD)^14^. Animal studies indicated that the anti-obesity effect of BBR might relate to its pro-thermogenesis effect in mature adipocytes via AMPK-PGC-1α pathway^15^. However, to the best of our knowledge, the effect of BBR on human BAT mass and activity as well as its underlying mechanisms has not been fully understood.

In the present study, we demonstrated that BBR intervention concurrently increased BAT mass and activity, thereby contributing to a reduction in BW and improvement in insulin sensitivity in patients with NAFLD. Moreover, we unexpectedly found that, apart from the reported thermogenesis activation in mature adipocytes, BBR effectively promoted brown adipocyte differentiation both in vitro and in vivo. Our data support that chronic BBR intervention can recruit and activate BAT in humans, which might be a promising strategy for the treatment of obesity and related metabolic diseases.

## Results

### BBR intervention promotes BAT recruitment and activity in mildly overweight patients with NAFLD

**Fig. 1:**
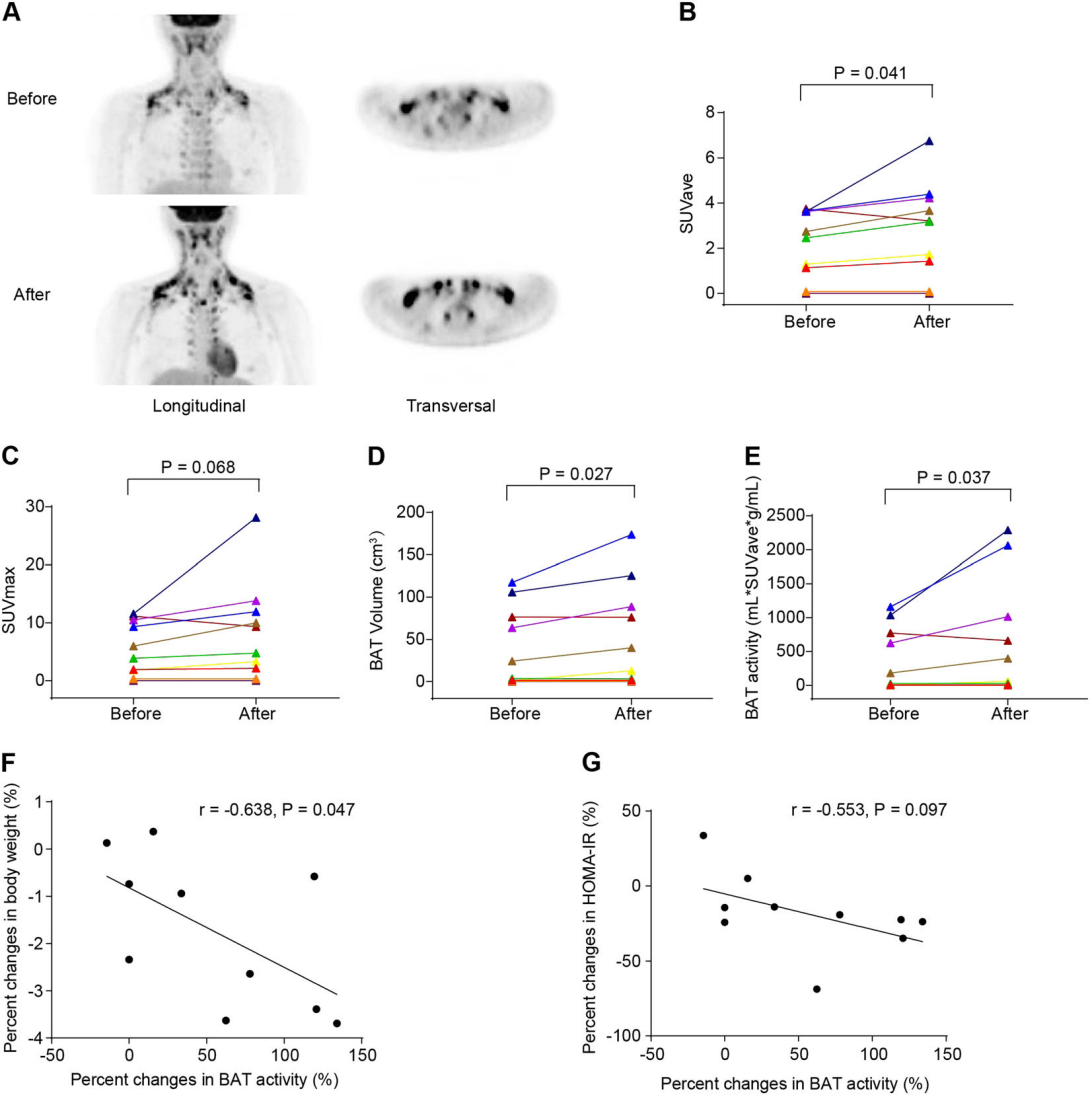
BBR increases the mass and activity of BAT in NAFLD patients. **a** ^18^F-FDG PET/CT images in representative subject of the 10 before and after BBR intervention. **b** Mean cold-activated standard uptake value (SUV) in BAT regions. **c** The maximal SUV in BAT regions. **d** Detectable BAT volume. **e** BAT activity as reflected by ^18^F-FDG uptake. **f** The linear regression of BAT activity change percentage and body weight change percentage after BBR intervention. **g** The linear regression of BAT activity change percentage and HOMA-IR change percentage after BBR intervention. *n* = 10. Data are expressed as mean ± SEM. *P < 0.05, **P < 0.01, ***P < 0.001 by paired Student’s *t* test

A total of 10 overweight individuals with NAFLD were recruited to undergo cold-activated ^18^F-FDG-PET/CT scanning before and after BBR administration for 1 month. During the follow-up period, the participants were required to maintain their previous exercise and dietary habits, and no exceptional events were reported. After 1-month BBR treatment, the mean standardized uptake value (SUV-mean), volume and activity of BAT were increased from 2.6 (0.8–3.6), 14.1 (0.9–83.9) cm^3^, and 103.1 (3.1–838.9) ml*SUVave*g/ml to 3.2 (1.1–4.3), 26.5 (1.3–98.0) cm^3^, and 228.2 (5.1–1276.5) ml*SUVave*g/ml, respectively (All P < 0.05) (Fig. 1a–e). Meanwhile, the SUV-mean in liver and heart were unchanged after BBR intervention (Table 1). In addition, the basal oral temperature of the subjects increased from 36.3 ± 0.3 °C to 36.5 ± 0.3 °C (*P* < 0.05), suggesting mildly enhanced heat production and energy expenditure (Table 1). Taken together, these results indicate that BBR intervention promotes both BAT mass and thermogenesis activity in humans.

**Table 1:** Comparison of metabolic parameters before and after BBR intervention

Parameters	Before (*n* = 10)	After (*n* = 10)	*P* value^a^
BW (kg)	71.2 ± 9.5	70.0 ± 9.6	0.011
BMI (kg/m^2^)	24.4 ± 2.0	24.0 ± 2.1	0.011
Waist circumference (cm)	81.7 ± 7.2	80.9 ± 6.9	0.076
Oral temperature (°C)	36.3 ± 0.3	36.5 ± 0.2	0.049
FBG (mmol/L)	5.1 ± 0.4	4.9 ± 0.3	0.194
Insulin (μU/mL)	11.3 ± 5.5	9.4 ± 4.5	0.053
HOMA-IR	2.6 ± 1.3	2.0 ± 1.0	0.030
Subcutaneous fat area (cm^2^)	155.1 ± 34.5	162.5 ± 36.3	0.126
Visceral fat area (cm^2^)	62.5 ± 22.9	58.2 ± 23.2	0.110
Visceral/subcutaneous ratio	0.41 ± 0.13	0.37 ± 0.14	0.026
Liver SUV-mean	2.22 ± 0.43	2.51 ± 1.10	0.452
Liver SUV-max	2.86 ± 0.32	3.22 ± 1.13	0.391
Heart SUV-mean	4.29 ± 2.16	3.88 ± 2.43	0.617
Heart SUV-max	5.41 ± 2.84	4.62 ± 2.92	0.475

^a^Paired two-tailed *t* test

### BBR intervention induces clinical metabolic improvements in NAFLD patients

Increased BAT activity is closely linked to metabolic improvement, such as weight loss, reduced inflammation, and increased insulin sensitivity^8–11^. As expected, 1-month BBR treatment significantly decreased BW (71.2 ± 9.5 vs 70.0 ± 9.6 kg, *P* = 0.011) and BMI (24.4 ± 2.0 vs 24.0 ± 2.1 kg/m^2^, *P* = 0.011) (Table 1). The visceral to subcutaneous fat area ratio by magnetic resonance imaging markedly decreased (0.41 ± 0.13 vs 0.37 ± 0.14, *P* = 0.026), accompanied by increased insulin sensitivity, indicated by reduced homeostasis model assessment for insulin resistance (HOMA-IR) (2.6 ± 1.3 vs 2.0 ± 1.0, *P* = 0.030) after BBR intervention (Table 1). The serum FBG and lipid profiles were in the normal range before and after BBR treatment, and showed no significant changes (Table 1).

Correlation analyses showed that the percentage changes in BW were negatively associated with the percentage changes in the BAT activity after BBR intervention (*r* = −0.638, *P* = 0.047) (Fig. 1f). In addition, there were a similar tendency between the changes in the HOMA-IR and the changes in the BAT activity (Fig. 1g). The serum levels of BAT activity-related endocrine factors^16^, including free thyroxine (T4), adrenocorticotropic hormone (ACTH), thyroid-stimulating hormone (TSH), epinephrine, irisin, and FGF21, were not changed after BBR intervention (Supplemental Table 1). Of note, BBR did not induce BAT activation in two subjects who had undetectable BAT activity at baseline (Supplemental Fig. 1), suggesting that 1-month BBR treatment was insufficient to promote brown adipose formation in the BAT-negative individuals.

Collectively, these data indicate that BBR promotes BAT recruitment and activation, which might partially contribute to weight loss and improvement of insulin sensitivity in humans.

### Chronic BBR treatment enhances BAT thermogenesis and energy expenditure in diet-induced obese (DIO) mice and chow-fed lean mice

**Fig. 2:**
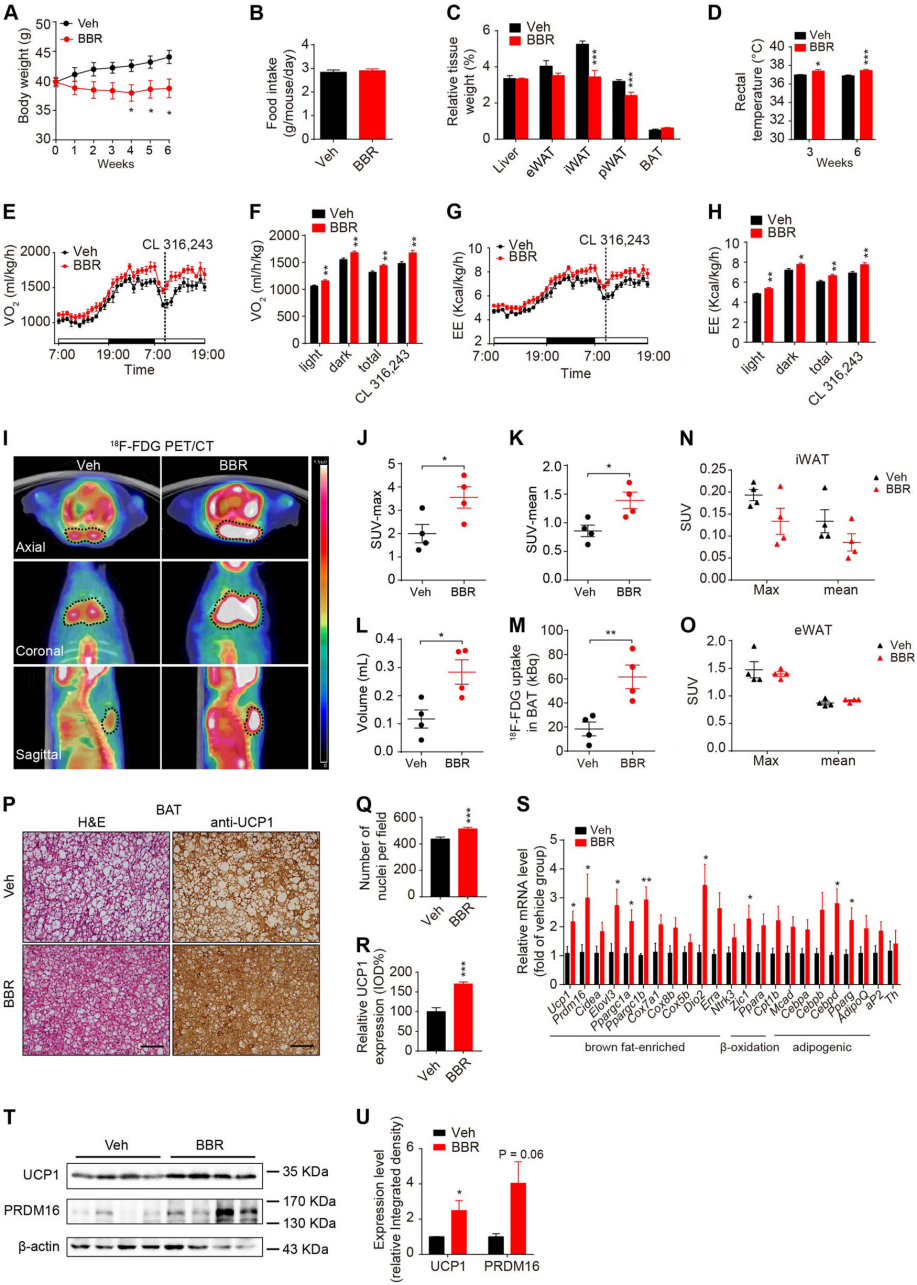
Chronic BBR treatment enhances BAT thermogenesis and reduces obesity in diet-induced obese (DIO) mice. **a**–**c** Body weight, food intake, and tissue weight of DIO mice after treatment. *n* = 7–8. **d** Rectal temperature of DIO mice. *n* = 7–8. **e**–**h** O_2_ consumption **e**–**f** and energy expenditure **g**–**h** of DIO mice under basal and CL 316,243-stimulated conditions. *n* = 7–8. **i–o** ^18^F-FDG PET/CT imaging of DIO mice: representative ^18^F-FDG PET/CT images with dashed lines marking the interscapular BAT region **i**; Maximal SUV **j**, mean SUV **k**, estimated BAT volume **l**, and total ^18^F-FDG uptake (kBq) **m** in BAT. The maximal and mean SUV in iWAT **n** and eWAT **o**. *n* = 4. **p**–**r** Representative H&E staining and UCP1 IHC images of BAT in DIO mice after treatment **p**; Quantification of nuclei number of brown adipocytes per field **q**. Relative UCP1 protein expression in BAT depicted by relative integrated optical density (IOD %) **r**. *n* = 7–8, 4–8 slides/mouse. **s** Relative mRNA levels of indicated genes in BAT after treatment for 17 days. *n* = 6. **t–u** Western blot analysis **t** and relative integrated density **u** of indicated protein in BAT of DIO mice after treatment. *n* = 7–8. Data are expressed as mean ± SEM. *P < 0.05, **P < 0.01, ***P < 0.001 compared with vehicle by Student’s *t* test

To mimic the above clinical trial in mouse model, the high-fat diet (HFD) induced obese (DIO) mice were subjected to BBR treatment for 6 weeks in order to further validate the long-term metabolic effects of BBR on BAT. BBR treatment significantly decreased BW and the wet weights of inguinal WAT (iWAT) and perirenal WAT without affecting food intake (Fig. 2a–c). BBR elevated the rectal temperature of DIO mice by ~0.4 °C and 0.6 °C in the 3rd and 6th week of treatment, respectively (Fig. 2d). As expected, BBR-treated animals exhibited higher O_2_ consumption, CO_2_ production, and energy expenditure compared with vehicle group at basal condition and in response to the stimulation of β3-adrenergic receptor agonist CL 316, 243, with unchanged locomotor activity at the beginning of the 4th week of treatment before the BW difference of the groups just started to show significance (Fig. 2e–h and Supplemental Fig. 2A–C). Notably, although the basal respiratory exchange ratio (RER) was unaffected by BBR treatment, but was decreased upon CL 316,243 stimulation (Supplemental Fig. 2D), indicating a shift of fuel preference from carbohydrates towards fatty acids upon BAT activation. Micro ^18^F-FDG PET/CT imaging was further used to assess the mass and activity of BAT of DIO mice after 6 weeks treatment (Fig. 2i). The maximal and mean SUV of BAT were significantly augmented in BBR-treated animals (Fig. 2j, k). As calculated, the volume of detectable BAT and total ^18^F-FDG uptake of BAT were markedly increased by BBR treatment (Fig. 2l, m). Meanwhile, the maximal and mean SUV in iWAT and eWAT remained unaltered (Fig. 2n, o), which suggests that BBR has no impact on the thermogenesis capacity of both iWAT and eWAT. Furthermore, BBR-treated animals displayed improved glucose metabolism and decreased plasma insulin level (Supplemental Fig. 2E–I and Supplemental Table 2).

As BBR could be distributed into different adipose tissue depots through i.p. injection (Supplemental Fig. 3), changes in gene expression and morphology of different adipose depots of DIO mice were analyzed to examine the effect of BBR on adipose tissues. The BAT of BBR-treated DIO mice displayed a more condensed morphology and the nuclei number of brown adipocytes were clearly increased compared with that of vehicle group (Fig. 2p, q), whereas BAT weight was comparable with vehicle group, indicating BBR treatment might stimulate the proliferation or differentiation of brown adipocytes in BAT. The mRNA levels of brown fat-enriched genes such as *Ucp1*, *Prdm16*, *Elovl3*, *Ppargc1a*, and *Ppargc1b* and pan-adipocyte markers such as *Cebpd* and *Paparg* in BAT of DIO mice was significantly upregulated upon BBR treatment (Fig. 2s). Moreover, the expression levels of thermogenic protein UCP1 and PRDM16 were markedly elevated in BAT after BBR treatment (Fig. 2r, t, u). In accordance with the ^18^F-FDG PET/CT result, BBR treatment did not induce browning characteristic in iWAT or eWAT (Supplemental Fig. 4). Taken together, these results indicate BBR specifically enhance BAT thermogenesis and increase energy expenditure in DIO mice.

In addition, we also verified the pro-BAT and pro-energy expenditure effect of BBR in age-matched chow-fed lean mice. The BW, tissue weight as well as food intake of the lean mice were not significantly altered after BBR treatment (Supplemental Fig. 5A–C). Although the basal rectal temperature was unchanged in both groups at room temperature (RT), BBR treatment notably increased the rectal temperature of lean mice housed at 4 °C (Supplemental Fig. 5D, E). Moreover, ^18^F-FDG PET/CT imaging revealed that BBR treatment enhanced thermogenic activity of BAT in lean mice (Supplemental Fig. 5F–H). BBR consistently increased O_2_ consumption, CO_2_ production, and energy expenditure without affecting RER and locomotor activity in lean mice (Supplemental Fig. 5I–Q). Also, the nuclei number of brown adipocyte and UCP1 expression level in BAT of lean mice were increased by BBR treatment (Supplemental Fig. 5R–T). Importantly, no significant changes in BW and glucose/lipid profiles were observed (Supplemental Table 2), suggesting that BBR-induced metabolic improvements in DIO mice might occur subsequent to enhanced BAT thermogenesis.

Overall, these data support that BBR treatment initially enhances BAT thermogenesis probably through facilitating BAT development, thereby promotes energy expenditure and improves glycolipid metabolism in DIO mice, validating previous results observed in humans.

### BBR promotes brown adipocyte differentiation in murine and human brown preadipocytes

**Fig. 3:**
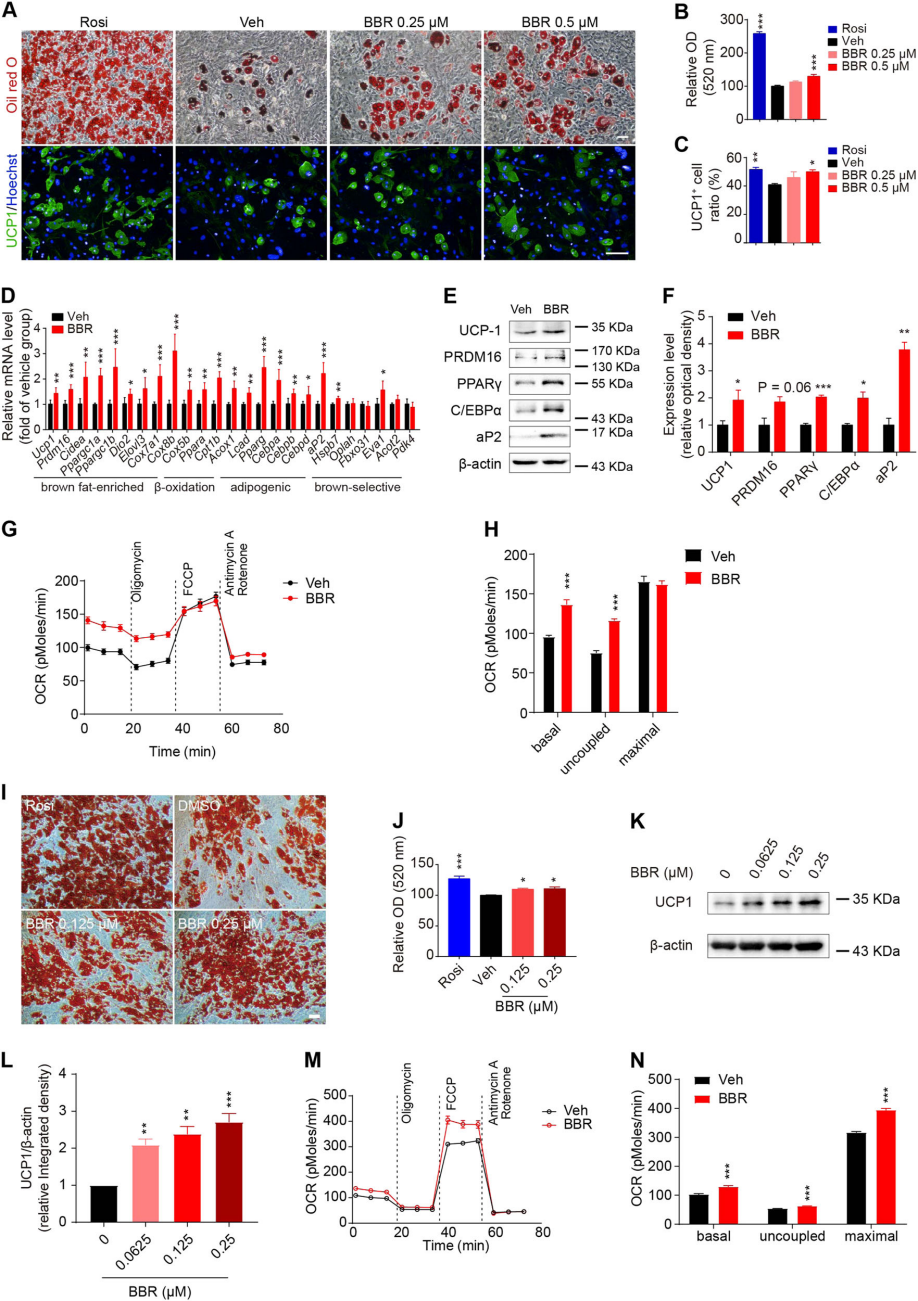
BBR promotes brown differentiation in both mouse and human brown preadipocytes. **a**–**h** BAT-SVF cells on day 8 of differentiation: representative oil red O staining and immunofluorescence staining of UCP1 (green) and Hoechst (blue) **a**, Rosiglitazone (Rosi) as a positive control; relative optical density (OD) measurement at 520 nm **b**; UCP1-positive cell ratio **c**; relative mRNA level of indicated genes (BBR 0.25 μM) **d**; western blot analysis of indicated proteins **e**–**f**, β-actin was used as loading control; Basal, uncoupled, and maximal OCR **g**–**h**. **i**–**n** Human fetal brown preadipocyte at day 14 of differentiation: representative oil red O staining images **i** and relative OD measurement at 520 nm **j**; representative western blot analysis of indicated protein levels **k**; relative integrated density of UCP1/β-actin were determined **l**. Basal, uncoupled, and maximal OCR **m**–**n**. Data are expressed as mean ± SEM. *P < 0.05, **P < 0.01, ***P < 0.001 compared with vehicle by Student’s *t* test or one-way ANOVA followed by Dunnett’s multiple comparisons for comparison of two or more groups

To demonstrate the effect of BBR on brown adipocyte differentiation in cell-autonomous fashion, primary stromal vascular fraction (SVF) cells isolated from BAT (BAT-SVF) were induced into differentiation in the presence or absence of BBR. Oil red O staining and UCP1 immunofluorescence staining showed that BBR increased lipid accumulation and the UCP1-positive cell ratio in differentiated BAT-SVF cells in a dose-dependent manner (Fig. 3a–c). BBR significantly upregulated mRNA levels of brown fat-enriched genes such as *Ucp1*, *Prdm16*, *Cidea*, *Ppargc1a*, *Ppargc1b*, *Elovl3*, *Cox7a1*, and *Cox8b*, fatty acid β-oxidation genes such as *Ppara*, *Cpt-1b*, *Acox1*, and *Lcad*, as well as adipogenic genes such as *Pparg*, *Cebpa* and *aP2* in differentiated BAT-SVF cells (Fig. 3d). Meanwhile, BBR also increased the expression levels of UCP1 and PRDM16, along with adipogenic markers such as PPARγ, C/EBPα, and aP2 (Fig. 3e, f). Furthermore, higher basal and uncoupled oxygen consumption rate (OCR) were observed in BBR-treated BAT-SVF cells at day 8 of differentiation (Fig. 3g, h), indicating that BBR positively regulates the brown adipocyte differentiation of BAT-SVF cells. Similar to the in vivo results of WAT, BBR had no significant influence on beige adipogenesis in SVF cells isolated from iWAT and eWAT (Supplemental Fig. 6).

Moreover, BBR significantly increased lipids content and UCP1-positive cell ratio in multipotent mesenchymal progenitor C3H10-T1/2 cells at day 8 of brown differentiation (Supplemental Fig. 7A–C). BBR treatment throughout the brown differentiation augmented the expression levels of brown fat-enriched genes and common adipogenic genes in C3H10-T1/2 cells (Supplemental Fig. 7D–G). Western blot analysis showed that BBR increased the expression of brown fat marker UCP1 and PRDM16 as well as the adipogenic factor PPARγ and C/EBPα in C3H10-T1/2 cells in a concentration-dependent manner (Supplemental Fig. 7H, I). In addition, BBR also promoted oxygen consumption in C3H10-T1/2 cells under basal and oligomycin-stimulated condition (Supplemental Fig. 7G–K). Taken together, these results indicate that BBR selectively promotes brown adipogenesis in brown preadipocytes and mesenchymal progenitors, but has no effect on beige adipogenesis in white preadipocytes from iWAT and eWAT.

To investigate whether BBR could affect brown adipocyte differentiation in human preadipocytes, primary human fetal brown preadipocytes were induced into differentiation and concurrently treated with BBR for 14 days. Oil O red staining shown that BBR slightly increased lipids accumulation and western blot analysis displayed that BBR significantly upregulated the expression level of UCP1 in a concentration-dependent manner in primary human brown preadipocytes at the end of differentiation (Fig. 3i–l). Furthermore, the basal, uncoupled, and maximal OCR of differentiated primary human brown preadipocytes were also increased by BBR treatment (Fig. 3m, n). These data indicate that BBR promoted brown adipogenesis in human fetal brown preadipocytes, which might contribute to the BBR-induced BAT recruitment in humans.

### PRDM16-mediated brown adipogenesis is essential for BBR-induced BAT thermogenesis in vivo

**Fig. 4:**
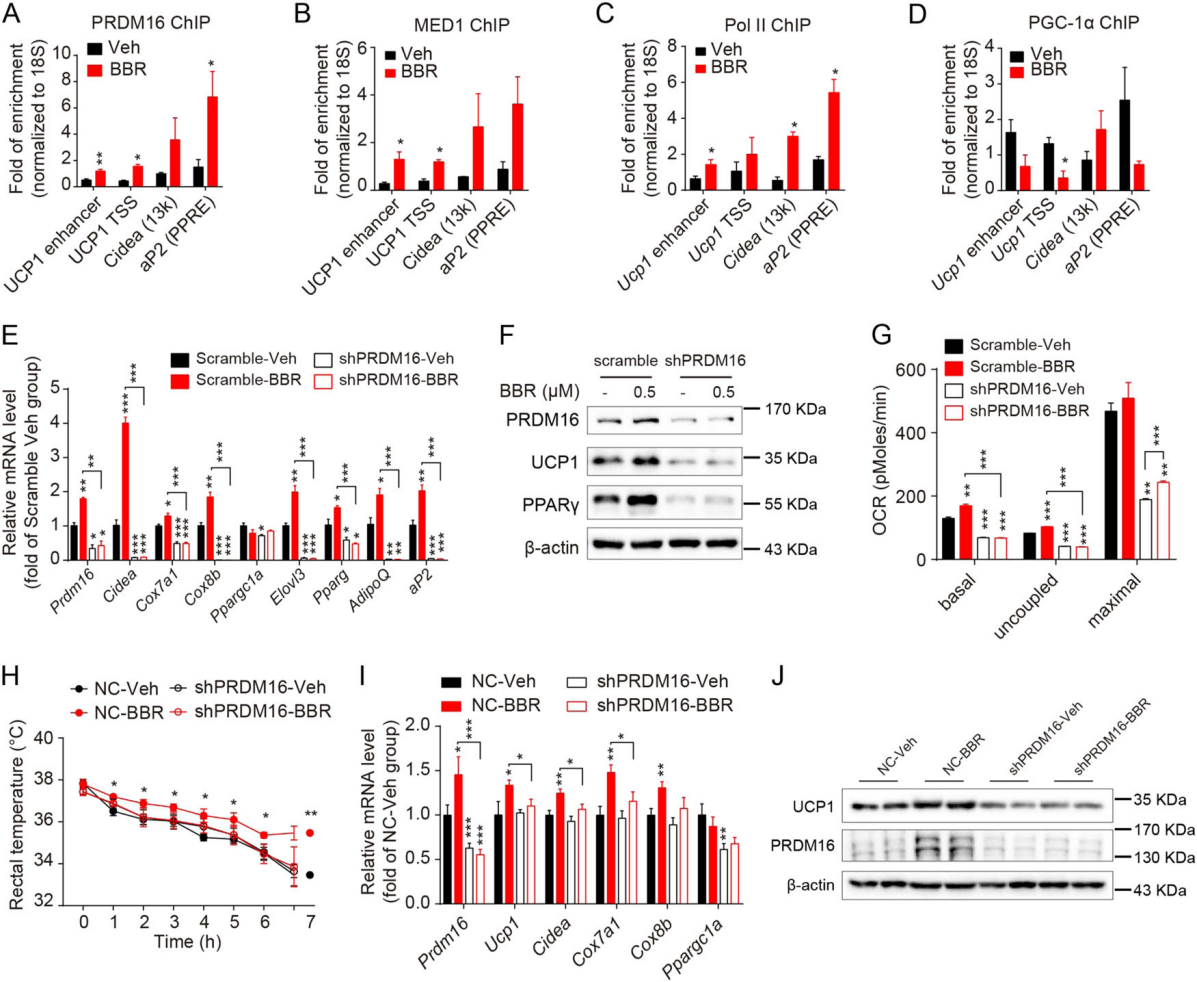
PRDM16 played an indispensable role in BBR-induced brown adipogenesis. **a**–**d** ChIP-qPCR analysis for PRDM16, MED1, PolII, and PGC-1α binding to indicated genes in differentiated C3H10-T1/2 cells on day 8 of differentiation. **e**–**g** The mRNA levels of the indicated genes **e**, representative western blot images of the indicated proteins **f** and average OCR **g** in scramble or shPRDM16 stable cells on day 8 of differentiation. **h** Rectal temperature of BAT-specific PRDM16 knockdown mice and WT mice (NC) at 4 °C for 7 h after 3 weeks treatment. *n* = 8–9. **i** The expression levels of brown fat-enriched genes in BAT of BAT-specific PRDM16 knockdown mice and NC mice after 6 weeks treatment. *n* = 8–9. **j** Western blot analysis and relative integrated density of indicated proteins in BAT of BAT-specific PRDM16 knockdown mice and NC mice after 6 weeks treatment. *n* = 4. Data are expressed as mean ± SEM. *P < 0.05, **P < 0.01, ***P < 0.001 compared with Veh or Scramble-Veh or NC-Veh by Student’s t test

The above findings had shown that PRDM16 expression was obviously induced by BBR treatment, hence eliciting brown adipogenesis both in vivo and in vitro. The binding of PRDM16, to the enhancer and transcription start site (TSS) region of *Ucp1* as well as the peroxisome proliferator-activated receptor region of *aP2* were markedly induced by BBR, accompanied by the increased recruitment of MED1, a component of mediator complex and also a co-activator of PRDM16^17,18^, as well as the positive control RNA Poll II (Fig. 4a–d). Whereas the binding of PGC-1α to these regions except for the *Ucp1* TSS remained unchanged by BBR, indicating that BBR-induced recruitment of PRDM16, but not PGC-1 α probably mediates the induction of brown fat transcription in C3H10-T1/2 cells.

To test this hypothesis, C3H10-T1/2 cell lines stably expressing shRNA-targeting PRDM16 (shPRDM16) or a scramble control were induced to undergo brown adipocyte differentiation in the presence or absence of BBR. QPCR testing and western blot analysis showed that the both mRNA and protein level of PRDM16 were substantially reduced in shPRDM16 cells, indicating successful PRDM16 knockdown cell model was achieved (Fig. 4e, f). Oil red O staining revealed that BBR enhanced lipid accumulation in control cells, but not in shPRDM16 cells, indicating that BBR-induced brown adipogenesis was dampened by PRDM16 knockdown (Supplemental Fig. 8A, B). The induction of UCP1 and PPARγ expression by BBR was robustly blunted in shPRDM16 cells (Fig. 4f and Supplemental Fig. 8C), and similar results were observed in the expression levels of other brown fat-enriched and adipogenic genes (Fig. 4e). The basal and uncoupled OCR in control cells were augmented by BBR, but this effect was not observed in shPRDM16 cells (Fig. 4g and Supplemental Fig. 8D). Collectively, these results demonstrate that BBR-facilitated brown adipogenesis is basically dependent on the existence of PRDM16 in vitro.

Furthermore, in order to examine the role of PRDM16 in BBR-promoted BAT thermogenesis in vivo, shPRDM16 lentivirus or scramble control lentivirus was injected into the interscapular fat pads of adult WT mice to generate a BAT-specific PRDM16 knockdown model (BAT-shPRDM16 mice) or negative control model (NC mice), which were subsequently administrated with saline or BBR for 6 weeks after 3 weeks of recovery. BAT-shPRDM16 mice exhibited increased weights of iWAT and eWAT and slightly decreased BAT weight compared with NC mice, with unaltered total fat mass and BW (Supplemental Fig. 8E–H). BBR treatment had no effect on the BW, body composition, and food intake in both NC mice and BAT-shPRDM16 mice. Cold exposure study showed that BBR treatment significantly elevated rectal temperature of NC mice at cold temperature, whereas did not impact that of BAT-shPRDM16 mice (Fig. 4h). This result suggests that sufficient PRDM16 expression in BAT is necessary for the pro-adaptive thermogenesis effect of BBR in vivo. QPCR analysis revealed that BBR treatment increased the mRNA levels of brown fat markers such as *Prdm16*, *Ucp1*, *Cidea*, and *Cox7a1* in BAT of control mice, whereas had no significant effect in that of BAT-shPRDM16 mice (Fig. 4i). Likewise, the expression levels of PRDM16 and UCP1 were notably increased upon BBR treatment in NC mice, whereas stayed unchanged in BAT-shPRDM16 mice (Fig. 4j and Supplemental Fig. 8I).

Taken together, these data indicate that apart from the mechanism of BBR that activates thermogenesis of mature adipocytes reported by Zhang et al.^15^ previously, PRDM16-mediated brown adipogenesis also has an indispensable role in BBR-induced BAT thermogenesis in vivo.

### BBR increases the cellular content of TCA metabolite α-Ketoglutaric acid (α-KG) and decreases the DNA methylation level of *Prdm16* promoter in brown preadipocyte

**Fig. 5:**
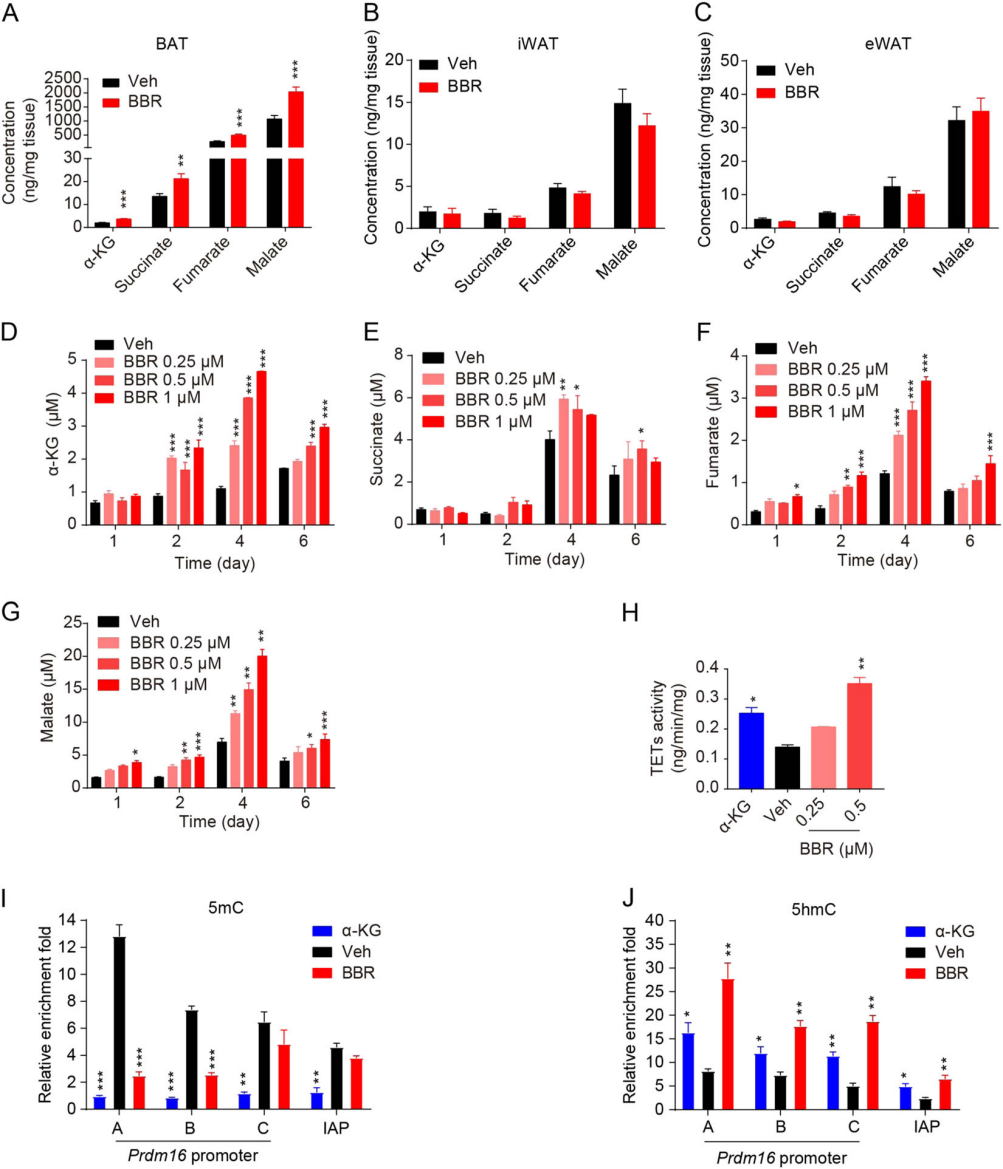
BBR epigenetically decreased the DNA methylation level of PRDM16 promoter. **a**–**c** The levels of α-KG, succinate, fumarate, and malate in BAT **a**, iWAT **b**, and eWAT **c** of DIO mice after 6 weeks treatment. *n* = 10. **d**–**g** The α-KG **d**, succinate **e**, fumarate **f**, and malate **g** level in C3H10-T1/2 cells during brown differentiation. **h** The activity of TETs in C3H10-T1/2 cells at day 4 of differentiation. **i**–**j** The enrichment levels of 5-mC **i** and 5-hmC **j** in *Prdm16* promoter of C3H10-T1/2 cells at day 4 of differentiation. Data are expressed as mean ± SEM. *P < 0.05, **P < 0.01, ***P < 0.001 compared with vehicle by Student’s *t* test or one-way ANOVA followed by Dunnett’s multiple comparisons for comparison of two or more groups

Given the pivotal role of PRDM16 in BBR-induced brown adipogenesis and thermogenesis, we further focused on illuminating the underlying mechanism of BBR in regulating PRDM16 expression. It is well established that BBR acts as a classic indirect AMPK activator by inhibiting the activity of complex I to improve gluco-lipid metabolism in rodent models^13–15^. Western blot analysis showed that acute treatment of BBR significantly activated AMPK signaling pathway in C3H10-T1/2 cells and BAT-SVF cells in a concentration-dependent manner (Supplemental Fig. 9A–D). Moreover, chronic BBR treatment induced AMPK activation in the BAT of chow-fed lean mice (Supplemental Fig. 9E, F). To be noted, it was reported previously that AMPKα ablation suppresses the active DNA demethylation of *Prdm16* promoter and brown adipogenesis through reducing cellular α-KG level^19^. Therefore, we wondered whether BBR as an AMPK activator could affect the concentration of α-KG and thus control the transcription of *Prdm16*. First, the levels of TCA intermediates including α-KG in the different adipose depots of DIO mice after 6 weeks treatment were determined by liquid chromatography tandem-mass spectrometry (LC-MS/MS). As a result, the levels of α-KG, succinate, fumarate, and malate were remarkably increased by chronic BBR treatment particularly in BAT, whereas these parameters sustained unaltered in iWAT and eWAT (Fig. 5a–c). In addition, BBR treatment elevated the levels of α-KG, fumarate and malate in C3H10-T1/2 cells since day 2 of differentiation, significantly at day 4 (Fig. 5d–g).

Given that α-KG is able to facilitate TET-mediated DNA demethylation, the effect of BBR on the TET activity and the enrichment of 5-methylcytosine (5-mC) and 5-Hydroxymethylcytosine (5-hmC) in Prdm16 promoter were further examined. The result revealed that BBR significantly enhanced the TET activity in C3H10-T1/2 cells at day 4 of differentiation (Fig. 5h). Meanwhile, the 5-mC level in the *Prdm16* promoter was significantly reduced and the 5-hmC level in the *Prdm16* promoter was increased upon BBR treatment for 4 days (Fig. 5i, j), indicating that BBR induced the active DNA demethylation in *Prdm16* promoter might be triggered by enhanced TET activity, thereby facilitating the *Prdm16* expression and ultimately promoting brown adipogenesis. Overall, these data demonstrated that BBR promotes PRDM16 expression through inhibiting the DNA methylation of *Prdm16* promoter, which might be caused by elevated α-KG production.

### AMPK activation is indispensable for BBR-induced brown adipogenesis and BAT thermogenesis

As the positive role of AMPKα in regulating brown adipocyte differentiation was reported previously^19^, it is necessary to address if AMPK activation by BBR really dominate its effect on brown adipogenesis in vitro. To this end, BAT-SVF cells were infected with sg_ *mAMPKα2-*cas9 and sg_*mAMPKα2*-cas9 lentivirus to knockdown both AMPKα1 and AMPKα2 (referred to as AMPKα-KD) before inducing into brown adipogenesis. BBR treatment throughout the differentiation significantly promoted brown adipogenesis in control BAT-SVF cells, with increased mRNA levels of brown fat markers and adipogenic genes, upregulated expression levels of UCP1 and PRDM16 and increased basal, uncoupled and maximal OCR, whereas had no effects on AMPKα-KD cells (Supplemental Fig. 10E–G). These results indicate that BBR-induced AMPK activation is required for promoting brown adipogenesis in vitro.

**Fig. 6:**
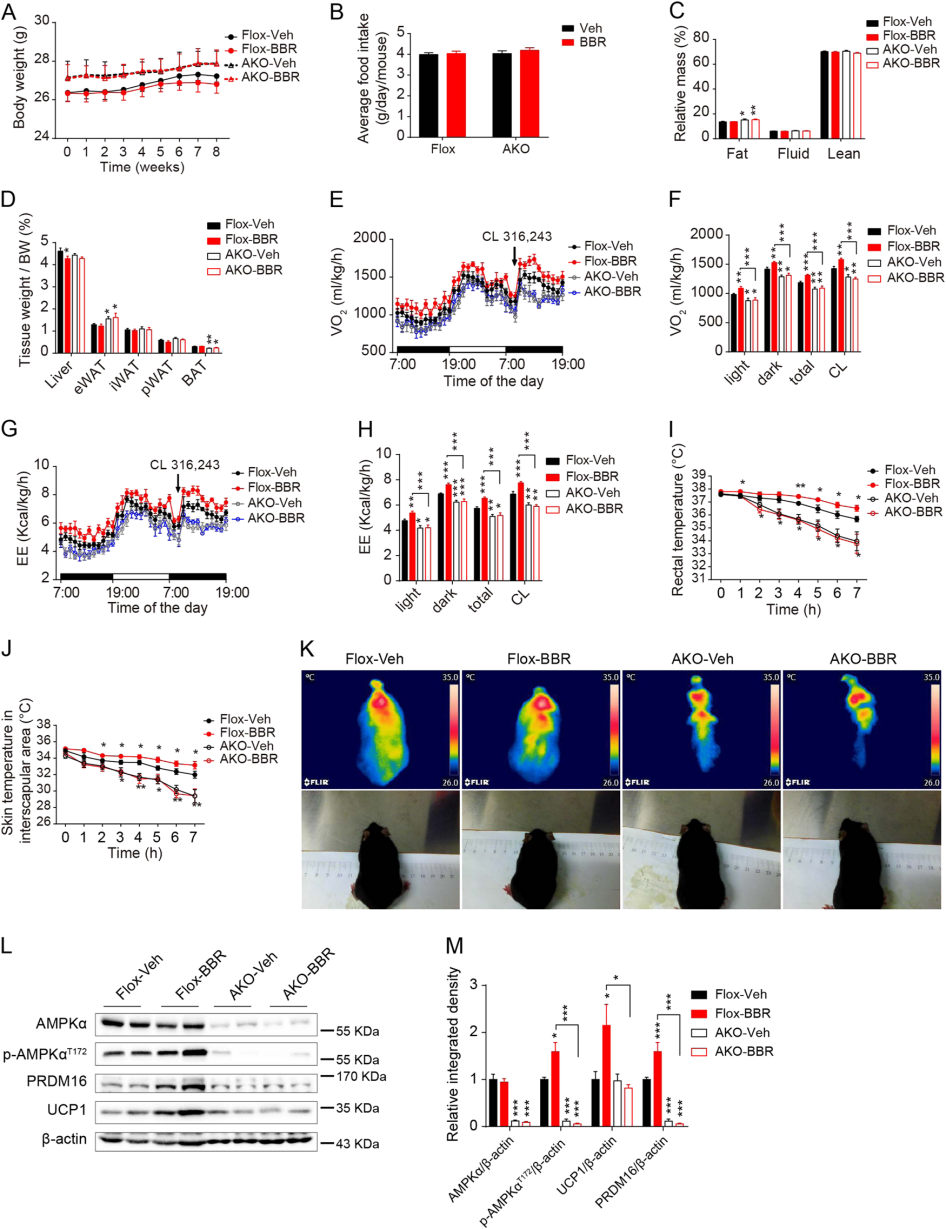
BBR-promoted brown adipogenesis and thermogenesis were depended on AMPK signaling. 13 weeks-old male AKO mice and floxed mice were treated with saline or BBR for 8 weeks. **a**–**b** Body weight **a** and food intake **b** were monitored during the treatment. *n* = 9–10. **c** The relative fat, fluid, and lean mass of the AKO and floxed mice after 8 weeks treatment. *n* = 9–10. **d** Relative weight of liver and different adipose depots of AKO mice and floxed mice after 8 weeks treatment. *n* = 9–10. **e**–**h** The VO_2_ change **e**, average VO_2_ **f**, EE change **g**, and average EE **h** of AKO and floxed mice after 4 weeks treatment under basal condition and after single injection with CL 316,243. *n* = 8. **i**–**k** The rectal temperature **i**, interscapular skin temperature **j** and representative thermal images of AKO and floxed mice housed at 4 °C for 7 h **k** after 6 weeks treatment. *n* = 9–10. **l**–**m** Representative western blot images **d** and the relative integrated density **e** of indicated proteins in BAT of AKO and floxed mice after 6 weeks treatment. *n* = 5–6. Data are expressed as mean ± SEM. *P < 0.05, **P < 0.01, ***P < 0.001 compared with Flox-Veh group by Student’s *t* test

In order to further confirm the necessity of AMPK activation for BBR’s effect on BAT thermogenesis and energy expenditure in vivo, adiponectin-Cre-driven adipose-specific AMPKα1/2 knockout mice (AKO) and age-matched floxed littermates were administrated with BBR or saline for 8 weeks. In line with our historical results^20^, AKO mice displayed increased fat mass, cold intolerance and decreased energy expenditure without any significant change in body eight and food intake compared with the floxed mice (Fig. 6a–i). The BW, fat mass, or tissue weight stayed unaltered after chronic BBR treatment (Fig. 6a–d). Metabolic studies revealed that BBR treatment remarkably elevated O_2_ consumption, CO_2_ production, and EE in floxed mice under basal condition and upon CL 316,243 stimulation, but did not affect that of AKO mice (Fig. 6e–h and Supplemental Fig. 10A–D), indicating the critical role of adipocyte AMPKα in BBR-promoted systemic energy expenditure. Furthermore, chronic BBR treatment notably elevated the rectal temperature as well as the skin temperature in the interscapular region of floxed mice upon cold challenge, but had no impact on that of AKO mice (Fig. 6i–k), indicating that the existence of AMPKα is necessary for BBR-promoted adaptive thermogenesis of BAT. Western blot analysis showed that the expression levels of UCP1 and PRDM16 in BAT of floxed mice but not in AKO mice were upregulated by chronic BBR treatment (Fig. 6l, m), which indicates that AMPK activation is essential for BBR-facilitated brown adipogenesis and thermogenesis in vivo.

Altogether, these data support that BBR promotes brown adipogenesis and BAT thermogenesis via activating AMPK signaling pathway.

## Discussion

The global prevalence of obesity and its comorbidities brings great challenge and demand for developing effective therapeutic strategies. The identification of thermogenic BAT in adult human has ignited intense interest and efforts to explore the novel anti-obesity approaches targeting brown and beige fat owing to their capacity of dissipating extra energy as heat. However, pharmacological agents aiming to increase the mass or activity of BAT, such as adrenergic receptor agonist L796568, Mirabegron and the thiazolidinediones Rosiglitazone, often have limited efficiency or unacceptable side effects in humans^21–25^. In the previous clinical studies, we reported that the BW of NAFLD patients were significantly reduced upon chronic BBR intervention, whereas the mechanism of which is still ambiguous. Here, we showed that BBR promoted brown adipogenesis in human fetal brown preadipocytes and induced BAT recruitment in NAFLD patients, providing the direct evidence that BBR intervention might ameliorate adiposity and improve insulin sensitivity. Importantly, we demonstrated that BBR epigenetically facilitated brown adipocyte differentiation and BAT thermogenesis through AMPK–PRDM16 axis, which would shed new light on the mechanism of BBR in regulation of BAT function.

Previous studies show that BBR inhibits white adipocyte differentiation by repressing the expression of adipogenic factors such as PPARγ and C/EBPβ^26,27^. Several studies reveal that AMPK activation facilitates both brown and beige adipogenesis as well as thermogenesis of adipose tissues^28,29^. Here we reported that BBR selectively promoted brown adipogenesis via AMPK activation, whereas had no obvious effect on beige adipogenesis. As brown and white preadipocytes are derived from distinct developmental origins^30^, the diversity of the transcriptional regulation networks might lead to the discrepancy of BBR’s effects. We found that PRDM16 was indispensable for BBR-induced brown adipogenesis and BAT thermogenesis and the expression level of PRDM16 usually differs in distinct fat depots. It has been reported that the PRDM16 expression level in the interscapular BAT is much higher than that in iWAT and eWAT owing to their intrinsic characteristics^6^. Moreover, according to our previous study experience, the inherent activation level of AMPK signaling pathway in different adipose depots varies in a certain manner. Similar to PRDM16 level, the inherent activation level of AMPK in BAT is higher than that in WAT (data not shown), which might be relevant with the different abundance of sympathetic nerves distribution^31^. Notably, AMPK activation in WAT of BBR-treated animals was not as apparent as in BAT (data not shown), which might help explain the favorable effect of BBR on thermogenesis of BAT rather than WAT and also the preference of promoting brown adipogenesis over beige adipogenesis by BBR treatment. As for the mechanism of BBR, we demonstrated that BBR epigenetically acted through AMPK-α-KG-PRDM16 axis to promote brown adipogenesis and BAT thermogenesis. It is worth mentioning that the significant elevation of TCA intermediate succinate was also observed following BBR treatment in vitro and in vivo. Recently, Mills et al*.*^32^ reported that succinate activates adipose tissue thermogenesis as an independent metabolic pathway, which lead to the hypothesis that in addition to α-KG, succinate might also play an unknown role in BBR-induced BAT thermogenesis.

In summary, we demonstrated for the first time that BBR administration increases both the mass and thermogenesis activity of BAT in humans, in parallel with a series of metabolic improvements. In addition, we found that BBR promotes brown adipocyte differentiation and BAT thermogenesis epigenetically through AMPK–PRDM16 axis, contributing to elevated systemic energy expenditure. BBR has already been widely demonstrated as an effective treatment for diarrhea, and these findings show promising potential for the clinical application of BBR for combating obesity and related metabolic disorders.

## Methods

### Plasmids and reagents

Lentiviral plasmid pGMLV-SC5 shPRDM16 was constructed by Genomeditech Company (Shanghai, China). Plasmid Cas9-GFP_sg_mAMPKα1 (#79004) and Cas9-GFP_sg_mAMPKα2 (#79005) were obtained from Addgene. Antibody sources are as follows: UCP1 (Alpha Diagnostic, UCP11-A) (for WB), UCP1 (Abcam, ab10983) (for immunohistochemistry (IHC)), PRDM16 (Abcam, ab106410), PPARγ (Santa Cruz, sc7273), PGC-1α (Calbiochem, ST1202), β-actin (Abgent, AM1021B) and normal rabbit IgG (Cell Signaling, #2729). CL 316,243, rosiglitazone, dexamethasone, 3-isobutyl-1-methylxanthine (IBMX), indomethacin, 3,3′,5-triiodo-l-thyronine (T3), polybrene, oligomycin, carbonyl cyanide 4-(trifluoromethoxy) phenylhydrazone (FCCP), rotenone, antimycin A and PMSF were purchased from Sigma-Aldrich. Recombinant human insulin (Eli Lilly) was purchased from Changzheng Hospital (Shanghai, China). The following ELISA kits were used for measurement of parameters in mouse plasma and human serum: human FGF21 (Millipore, #EZHFGF21–19K), human & mouse irisin (Phoenix, EK-067-29), human epinephrine (CUSBIO, CSB-E08677h), human ACTH (CUSBIO, CSB-E06873h), lactic acid (Reebio, 10010-717), NFEA (WAKO, 294-63601), mouse insulin (Millipore, EZRMI-13K), mouse leptin (Millipore, EZML-82K), mouse HDL-C (XinJianKangCheng Bio, E0303), mouse LDL-C (XinJianKangCheng Bio, E0403), mouse TG (Shanghai Fosun Long March, 1.02.1801), and mouse TC (Shanghai Fosun Long March, 1.02.0401).

### Subjects

Twelve mildly overweight NAFLD patients (age 29.7 ± 2.9 years) were recruited from the out-patient Department of Endocrinology, Zhongshan Hospital from December 2014 to February 2015 and December 2015 to February 2016. Because BAT activity is reversibly modulated by ambient temperature and remains stable during periods with small variations in ambient temperature^33,34^, we enrolled study participants from December to February to minimize the interference of ambient temperature based on historical weather records from Shanghai, China. The monthly mean temperature during the whole study was in the range of 9–11 °C (Supplemental Fig. 11). No participants had known acute or chronic diseases except for overweight, obesity, dyslipidemia, or NAFLD based on medical history, physical examination, and laboratory tests conducted in the clinic (including blood cell count, liver and renal function, blood glucose, lipid profile, thyroid hormone, hepatitis B and C antibodies, and autoimmune hepatitis antibodies). Subjects treated with anti-obesity drugs, adrenergic drugs, thyroid hormone, or glucocorticoid within 4 weeks before enrollment were excluded from the study. Ten participants finished the follow-up examination, and two withdrew from the study after the first cold-activated PET/CT scanning.

### Clinical study design

All subjects underwent cold-activated ^18^F-FDG PET/CT scanning as described by van Marken Lichtenbelt WD et al.^8^ and were then treated with BBR for 1 month. During the treatment, the subjects were required to maintain their previous exercise and dietary habits, and took BBR at a dosage of 0.5 g, 30 min before eating, three times a day, which was proved to be effective and safe in our previous study^14^. The subjects were required to participate in another cold-activated ^18^F-FDG PET/CT 1 month later. Both at the beginning and completion of the treatment, each participant received a face-to-face interview by a trained investigator, an assessment of anthropometric parameters and blood examinations for blood glucose, lipid profile, insulin, thyroid hormone, ACTH, glucagon, non-esterified fatty acid (NEFA), lactic acid, epinephrine, irisin, and FGF21 concentrations.

### Clinical measurements

Serum glucagon and leptin were measured using a Human Premixed Multi-Analyte Kit (R&D Systems, cat. LXSAHM) according to the manufacturer’s instructions (H-Wayen Biotechnologies). Levels of FBG, insulin, TG, TC, HDL-C, LDL-C, free T4 and TSH were determined using standard procedures at Zhongshan Hospital. Serum ACTH, epinephrine, NEFA, lactic acid, irisin, and FGF21 were measured according to manufacturer’s protocols.

### Cold-activated ^18^F-FDG PET/CT scanning

^18^F-FDG PET/CT scanning was performed from ~8:00 am to 11:00 am after an overnight fasting of at least 10 h. Before being positioned in the scanner, the subjects spent 2 h in a climate chamber kept at 16 °C while wearing light clothing. After 1 h of exposure to cold, the PET tracer ^18^F-FDG was administered intravenously at a dose of 0.13 mCi per kilogram BW, and scanning was performed after the second hour of exposure to cold. Six to seven bed positions were scanned for each subject, and each bed position took 3 min. Two nuclear-medicine physicians interpreted the PET-CT images using PMOD software (version 2.85). BAT activity and volume were quantitatively measured in the region of interest (ROI) by auto-contouring BAT areas with a set threshold^9^.

### Animal studies

Six-week-old male C57BL/6J mice (Shanghai SLAC Laboratory Animal Co., Shanghai, China) were housed in a temperature- and relative humidity-controlled room (22 ± 2 °C, 55 ± 5%) with a 12 h light–dark cycle and free access to food and water. For chronic anti-obesity studies, mice were fed a HFD starting at 6 weeks of age (60% calories from fat, 20% calories from protein, 20% calories from carbohydrate; Research Diets). At 14 weeks of age, HFD-fed mice (DIO) and chow-fed lean mice were randomly assigned to treatment groups and were i.p. administrated with saline or BBR (1.5 mg/kg/day) daily for 6 weeks between 9:30 and 10:30 am. BW and food intake were recorded daily. Rectal temperature was measured between 9:30 and 10:30 am using a rectal probe attached to a digital thermometer (BAT-12 Microprobe Thermometer; Physitemp, Clifton, NJ). Metabolic analysis using indirect calorimeter was conducted at the beginning of the 4th week of treatment. A glucose tolerance test (GTT) and metabolic analysis were performed in the 4th week of treatment. An insulin tolerance test (ITT) was conducted, and blood samples were collected in the 5th week of the study. Micro ^18^F-FDG PET/CT imaging was performed in the 6th week of treatment. At the end of the study, tissues were dissected, weighed, immediately frozen in liquid nitrogen, and stored at −80 °C. Plasma TC, TG, LDL, HDL, insulin, leptin, and irisin were measured using corresponding kits according to the manufacturers’ instructions.

Adipose-specific AMPKα1/α2 KO mice (AKO) were generated by mating AMPKα1/α2 floxed mice and Adiponectin-Cre mice (stock No: 010803, Jackson Laboratory, Bar Harbor, Maine, USA) as described previously^20^. The AKO mice and AMPKα1/α2 floxed mice were fed with chow diet and administrated with saline or BBR (1.5 mg/kg/day) for 8 weeks from 13 weeks of age. BW and food intake were recorded daily. Metabolic analysis was performed in the 4th week of treatment and cold exposure study was conducted in the 6th week of treatment. The body composition analysis was performed in the 8th week of treatment using ^1^H-nuclear magnetic resonance spectroscopy (Minispec LF90 II, Bruker).

To generate BAT-specific NC or shPRDM16 mice, high titer NC or shPRDM16 lentivirus were directly injected to the interscapular BAT pads of anesthetized 8 weeks-old wild type (WT) mice according to the method described previously^35^. After 3 weeks following the infection, the mice were administrated saline or BBR (1.5 mg/kg/day) daily for 6 weeks. Cold exposure study was performed in the 3rd week of treatment.

At the end of the studies, the tissues were dissected, weighed, immediately frozen in liquid nitrogen and stored at −80 °C.

### Metabolic analysis for animal study

O_2_ consumption, CO_2_ production, and locomotor activity were measured using a 16-chamber indirect calorimeter (TSE PhenoMaster, TSE system) according to the manufacturer’s instructions. The last administration was given 4 h before the experiment, and the mice were acclimated in the chambers for 24–28 h. Basal metabolic parameters were measured during the next 24 h, and CL 312,643-stimulated metabolic parameters were measured for 10 h after a bolus i.p. injection of CL 312,643 (1 mg/kg) in DIO mice. For chow-fed lean mice, basal metabolic parameters were measured for 48 h. The mice were maintained at 24 °C under a 12 h light–dark cycle. Food and water were available *ad libitum*. Locomotor activity was derived from *x* axis and *y* axis beam breaks, monitored every 17 min. O_2_ consumption, CO_2_ production, heat production, and RER were calculated as described previously^36^.

### ipGTT and ITT assays

Mice were fasted for 6 h and received a bolus i.p. injection of glucose (2 g/kg) or recombinant human insulin (0.75 U/kg). Tail-vein blood was collected for glucose concentration measurement before and 15, 30, 60, 90, and 120 min after glucose or insulin injection.

### Micro ^18^F-FDG PET/CT imaging

Micro ^18^F-FDG PET/CT imaging was performed on an Inveon MM Platform (Siemens Preclinical Solutions, Knoxville, Tennessee, USA) at the Shanghai Jiao-tong University Med-X Ruijin Hospital Micro PET/CT Research Center. DIO mice were fasted overnight and anesthetized via intravenous injection of pentobarbital sodium (10 mg/kg). The mice were then administrated ^18^F-FDG (150 μCi) 10 min after the anaesthetization and subjected to PET/CT analysis 40 min after the injection of the radiotracer. The PET/CT imaging protocols and image reconstruction and quantitative evaluation procedures used in this experiment were described previously^15^. The total accumulated amount of ^18^F-FDG in a ROI on micro PET images was calculated according to the following formula^37^:

$${\text{FDG}}\,{\text{uptake}}\,({\text{kBq}}) = \frac{{{\text{SUV}}_{mean}\left( {\frac{\text{g}}{\text{mL}}} \right) \times {\text{radiotracer}}\,{\text{activity}}\,(\mathrm{\mu} {\text{Ci}}) \times \left( {\frac{{\text{kBq}}}{\mathrm{\mu} {\text{Ci}}}} \right) \times {\text{ROI}}\,{\text{volume}}\,({\text{mL}})}}{{\text{Body}}\,{\text{weight}}\,({\text{g}})}$$

### Cold exposure

Mice were individually housed at 4 °C for 7 h without food but with free access to water. Rectal temperature was measured every hour with a BAT-12 microprobe digital thermometer and RET-3 mouse rectal probe (Physitemp Instruments, Clifton, USA). Thermo-images were taken using an E6 Thermal Imaging Infrared Camera (FLIR Systems) and the skin temperature were analyzed using FLIR Tools software.

### Histological analysis

Tissues were dissected and fixed in 4% neutral buffered formalin overnight and then placed in 70% ethanol for long-term storage. Paraffin processing, embedding, sectioning, H&E staining, and IHC were performed at Shanghai ZuoCheng Bio Company (Shanghai, China) according to standard protocols. The following antibodies and concentrations were used in the IHC assay: UCP1 antibody (1:50, Abcam) and peroxidase AffiniPure Goat Anti-Rabbit IgG (H + L) (1:200, Jackson ImmunoResearch). All slides were observed using a Leica DM6 B microscope at the indicated magnification, and images were captured with a sCMOS camera under the same parameter settings. Quantification of IHC images was performed using Image-Pro Plus software (Media Cybernetics Inc., Silver Springs, MD, USA).

### Tissue distribution assay

DIO mice were i.p injected with 5 mg/kg BBR. After 1 h, the animals were sacrificed and plasma, liver, different adipose depots, and muscle were collected and preserved at −80 °C. Tissue samples were analyzed with LC-MS/MS system (Agilent 1200 HPLC coupled to Agilent 6460 Triple Quad instrument, Agilent Technologies, USA) to detect the concentration of BBR. Samples were disrupted with methanol before analysis. The chemicals were firstly separated on Luna PFP (50 × 2.0 mm, 5 μm, Phenomenex) using mixture of methanol-0.1% formic acid and 5 mmol/L ammonium acetate solution (55:45, v/v) with following rate (0.65 ml/min, 0–5 min RT). The chemicals with m/z 336 and 320 were analyzed using electrospray ionization mode with capillary voltage setting to 4 kV. The drying gas temperature was 350 °C with a flow rate of 10 L/min, and the sheath gas temperature was 350 °C with a flow rate of 11 L/min. Data were analyzed by MassHunter Quantitative Analysis (version B.02.01, Agilent Technologies).

### Cell culture and stable cell line selection

Human fetal brown preadipocytes were purchased from ZenBio (Lot# BP8314-102113) and were maintained with Brown Preadipocyte Medium (cat# BPM-1) at 37 °C in a 5% CO_2_ incubator. Once the cells reached 100% confluency, the differentiation was initiated by changing the media into Brown Adipocyte Differentiation Medium (cat# BDM-2). After incubating for 7 days, the cells were fed with Brown Adipocyte Maintenance Medium (cat# BAM-1) for additional 7 days. After 14 days since the differentiation induction, the cells should appear rounded with lipid droplets and be suitable for assays. BBR or rosiglitazone (a positive control) was added throughout the differentiation. Experiments were performed on day 14 of differentiation.

HEK293 cells and 293T cells were purchased from the American Type Culture Collection (ATCC). C3H10-T1/2 cells were a kind gift from Guang Ning. These two types of cells were maintained in Dulbecco's Modified Eagle Medium (DMEM) supplemented with 10% fetal bovine serum (FBS) (Gibco) and 1% penicillin/streptomycin (Invitrogen) at 37 °C in a 5% CO_2_ incubator. For lentivirus production, 293T cells were transfected with viral vectors as well as packaging plasmids pCMV-VSV-G (Addgene # 8454) and psPAX2 (Addgene #12260) using Lipofectamine 2000 (Invitrogen). Viral supernatant was harvested 48 and 72 h later, and the samples were pooled and filtered through a 0.45 μm strainer together. C3H10-T1/2 cells were infected with scramble or shPRDM16 lentivirus supplemented with 8 μg/ml polybrene for 8 h. Stable transfected cells were selected with puromycin (2 mg/ml). The shRNA construct was a pGMLV-SC5 RNAi vector (Genomeditech). The target sequence used for the lentiviral shRNA expression vector targeting PRDM16 was 5′-GCAGTGACTTTGAGGATATCA-3′. At 48 h after reaching 100% confluency, C3H10-T1/2 cells were induced to undergo adipocyte differentiation by treating with DMEM containing 10% FBS, 0.5 mM IBMX, 125 nM indomethacin, 1 μM dexamethasone, 850 nM insulin, and 1 nM T3. Two days after induction, the cells were switched to maintenance medium containing 10% FBS, 850 nM insulin and 1 nM T3 for 8 days.

### Isolation of adipose SVF cells and in vitro differentiation

SVF cells from 6- to 7-week old male C57BL/6J mice were isolated as described previously^5^. In brief, adipose tissue was minced on ice and digested with 10 mg/ml collagenase D (Roche) and 2.4 mg/ml dispase II (Roche) in phosphate-buffered saline (PBS) supplemented with 1% penicillin/streptomycin for 45 min at 37 °C, followed by quenching with complete medium and filtering through a 100 μm strainer (BD Biosciences). The cell suspensions were centrifuged, suspended and filtered through a 40 μm strainer (BD Biosciences) and then further centrifuged and suspended before plating onto 10 cm dishes. SVF cells were cultured in DMEM/F12 supplemented with 10% FBS and 1% penicillin/streptomycin (Invitrogen). Adipocyte differentiation was carried out by treating confluent cells in growth medium supplemented with 0.5 mM IBMX, 125 nM indomethacin, 1 μM dexamethasone, 850 nM insulin, and 1 nM T3 for 48 h. Following this, the cells were maintained in growth medium supplemented with insulin and T3 for 8 days. To analyze the effects of BBR on differentiation, BBR, or rosiglitazone (a positive control) was added throughout the differentiation. Experiments were performed on day 8 of differentiation.

### Oil red O staining

Cells were washed twice with PBS and fixed with 4% paraformaldehyde for 30 min at RT, followed by incubation with Oil Red O (BBI Life Sciences) for 60 min. After staining, the cells were washed with PBS 2–3 times before being viewed under a light microscope for image capture. Absorbance was measured with a Spectra Max M5 (Agilent technologies) at 520 nm. The relative intracellular lipid content was expressed as the optical density at 520 nm relative to vehicle control (AU, arbitrary units).

### Quantitative RT-PCR analysis

Total RNA was isolated from cells or homogenized tissues using TRIzol reagent (Invitrogen). Complementary DNA was prepared from 1 μg of total RNA using PrimeScript Reverse Transcriptase (TaKaRa) according to the manufacturer’s instructions. After a 10-fold dilution, the cDNA was amplified using 2× SYBR Green qPCR Master Mix (Biotool) and a Stratagene Mx3005P (Agilent Technologies). Expression data were normalized to 36b4 (for cells) or 18S (for tissues). The sequences of the primers used in this study are listed in Supplementary Table 3.

### Western blot analysis

Differentiated cells were lysed with RIPA lysis buffer (Beyotime) containing 1 mM phenylmethylsulfonyl fluoride (PMSF) on ice and clarified by centrifugation at 12,000 g for 10 min at 4 °C. The protein content of the supernatants was determined using a BCA Protein Assay kit (ThermoFisher Scientific). Equal amounts of protein were electrophoresed through sodium dodecyl sulfate polyacrylamide gel electrophoresis after boiling for 10 min in loading buffer and then transferred to Immobilon-NC membranes. The membranes were blocked with 5% skim milk at RT for 1 h and incubated with the indicated antibodies overnight at 4 °C, followed by incubation with a secondary antibody at RT for 1 h. Protein expression was detected with enhanced chemiluminescence (GE Healthcare) using the ChemiDoc MP Imaging System (Bio-Rad).

### Immunofluorescence staining

Cells were washed with PBS and fixed with 4% paraformaldehyde for 30 min at RT. After permeabilizing with 1% Triton X-100 for 15 min on ice, the cells were blocked with 5% BSA for 1 h at 37 °C, and then incubated with the indicated primary antibodies at 4 °C overnight. After thorough washing, the cells were incubated with secondary antibodies conjugated with Alexa Fluor 488 (2 mg/ml, ThermoFisher Scientific, A-11034) at a 1:2000 dilution at RT for 1 h. Nuclei were stained with Hoechst 33342 (10 μg/ml, ThermoFisher Scientific, H3570) at a 1:2000 dilution for 5–10 min. Images were captured using an Operetta High Content Screening System (PerkinElmer) and analyzed with Columbus software (PerkinElmer).

### OCR measurement

Cells were plated in an XFe 96-well microplate (Seahorse Bioscience) and allowed to grow to confluency. Then, they were induced to undergo brown adipogenesis for 8 days, followed by OCR measurement at 37 °C using an XF24 analyzer (Seahorse Bioscience) according to the manufacturer’s instructions. Following this, 1 μM oligomycin, 2 μM FCCP, and 1 μM rotenone/antimycin A were used to detect uncoupled respiration, maximal respiration, and non-mitochondrial respiration, respectively.

### Nuclear extraction and TET activity assay

C3H10-T1/2 cells were induced into brown adipocyte differentiation and treated with α-KG or BBR at different concentrations throughout the differentiation. The cells nuclei were isolated by using Nuclear and Cytoplasmic Extraction Reagents (Beyotime, P0027) at day 4 of differentiation. TET activity was determined by using TET Hydroxylase Activity Quantification Kit (Fluorometric) (Abcam, ab156913) according to the manufacturer’s instructions.

### Hydroxymethylated and methylated DNA Immunoprecipitation ((h)MeDIP)

According to the method described previously^38^, in brief, genomic DNA of C3H10-T1/2 cells at day 4 of differentiation was isolated using the DNeasy Blood & Tissue kit (Qiagen, 69506). Genomic DNA (20 μg) was diluted in 300 μL TE buffer and sonicated by Bioruptor Waterbath Sonicator for 12 cycles 30 s on/30 s off, until the size of DNA was 300–1000 bp. The sonicated DNA was purified and 10% of the sonicated DNA was used as Input sample. The sonicated DNA (4 μg) was diluted in 450 μL TE buffer and subsequently denatured for 10 min at 95 °C and then immediately cooled on ice. The denatured DNA solution was diluted by adding 50 μL 10× IP buffer (100 mM Na-Phosphate, pH 7.0, 1.4 M NaCl, 0.5% Triton X-100). Then 5-Hydroxymethylcytosine (Zymo Research, A4001) or 5-methylcytosine antibody (Zymo Research, A3001) was added the DNA solution with rotation overnight at 4 °C. The DNA–antibody mixture was further incubated with pre-blocked Pierce™ magnetic protein A/G (Thermo Scientific, 88802) for 2 h. The beads–antibody–DNA complex were washed three times with 1× IP buffer and responded in 250 μL digestion buffer (50 mM Tris HCl, pH 8; 10 mM EDTA; 0.5% SDS) and then digested with 3.5 μL proteinase K (20 mg/mL) by incubating at 50 °C for 3 h. The DNA was extracted using ChIP DNA Clean & Concentrator (Zymo Research, D5205) and the purified DNA as well as the Input sample were undergone Quantitative RT-PCR analysis using primers listed in Supplemental Table 4. Relative enrichment folds of indicated DNA regions were determined after normalization to Input and β-action promoter was used as a negative control. IAP (repeat) was used as a positive control.

### Tricarboxylic acid intermediates analysis of cells and adipose tissue

C3H10-T1/2 cells were cultured in 12-well plate and induced into brown adipocyte differentiation. Cells were digested with trypsin per well followed by centrifugation, and were lysed with 400 μL of 80% methanol/water (v/v) including internal standards for 20 min at 4 °C. About 200 μL of the cell lysis solution was air-dried and reconstituted in 100 μL of H_2_O. For tissue samples, ~50 mg adipose tissues were weighted and homogenized in 1 mL PBS buffer. 100 μL cell lysis solution or tissue homogenate were added into a 96-well plate and mixed with 50 μL of 1 M O-benzylhydroxylamine solution and 50 μL of 1 M 1-ethyl-3-dimethylaminopropyl carbodiimide) solution followed by agitation at RT for 1 h to facilitate derivatization. Afterwards 300 μL of ethyl acetate was added and the plate was agitated for 20 min followed by centrifugation at 1900 rpm for 5 min. The organic layer was taken to a new plate and dried using a stream of nitrogen and subsequently reconstituted in 150 μL of 50% methanol/water. The samples were firstly separated by UPLC system using a Waters Acquity UPLC BEH C18 (50*2.1 mm, 1.7 µm) with a two-solvent system (A: 5 mM NH_4_OAc and 0.1% formic acid in H_2_O; B: 0.1% formic acid in acetonitrile) and a flow rate of 0.5 mL/min and then analyzed by a LC–triple quadrupole mass spectrometer (AB Sciex,QTRAP® 6500).

### ChIP assay

ChIP was performed on differentiated C3H10-T1/2 cells on day 8 of differentiation using an EZ-ChIP kit in accordance with the manufacturer’s instructions (Millipore). In brief, DNA-containing cell lysates were sheared into fragments of 200–1000 bp by sonication after fixation with formaldehyde. For immunoprecipitation, equal aliquots of cell lysates were incubated with anti-PRDM16 antibody, anti-PGC-1α antibody, anti-PolII antibody (Millipore) or rabbit IgG at 4 °C overnight. Precipitated DNA was amplified by real time PCR in triplicate using the primers listed in Supplementary Table 4. Data were normalized to 18S and expressed as fold of enrichment.

### Study approval

The human study was approved by the ethics committee of Zhongshan Hospital, Fudan University and was conducted in accordance with the guidelines of the Declaration of Helsinki. Written informed consent was obtained from all participants. All animal experiments were performed according to a protocol approved by the Institutional Animal Care and Use Committee (IACUC) of Shanghai Institute of Materia Medica.

### Statistics

Previous study showed that log_10_ BAT activity was normally distributed with a standard deviation of 0.72^21^. Mirabegron-induced increase in log_10_ BAT activity by an average of 2.00 mL*SUVave*g/mL was associated with ~5 kg weight reduction in the first year^21^ and ~1 kg reduction in the first month^39^. In our prior study of BBR treatment in patients with NAFLD, 1-month BBR treatment at 0.5 g tid cause an average of 0.5 kg weight reduction^14^. Therefore, we predicted that 1-month BBR treatment would produce a mean log_10_ BAT activity of 1.00 mL*SUVave*g/mL with a SD of 0.72, assuming a linear correlation between the weight reduction and enhanced log_10_ BAT activity. We determined that 8 subjects would be needed to reject the null hypothesis that the change in BAT activity before and after BBR treatment is 0 in a paired comparison with α = 0.05 and power 80%. The rate of detection of cold-activated BAT was about 80–90%^40^, so we estimated that studying 10 subjects would yield sufficient subjects with detectable BAT activity for statistical analysis.

For statistical analysis in the clinical study, continuous variables are expressed as mean ± SD, except for skewed variables, which are presented as the median with the interquartile range given in parentheses. Paired *t* tests or Wilcoxon rank sum tests were used to compare continuous parameters before and after the intervention. For statistical analysis in the animal and cellular studies, data are represented as mean ± SEM and are from at least three independent biological replicates. Differences between two groups were analyzed with a two-tailed unpaired *t* test. Multiple comparisons versus the corresponding control groups were analyzed with one-way analysis of variance (ANOVA) followed by Dunnett’s multiple comparisons test. Two-way ANOVA was used to examine interactions between variables. *P* < 0.05 was regarded as statistically significant.

## Supplementary information

Supplemental data revised

Supplementary figures
