# Supplementary material for: Berberine promotes the recruitment and activation of brown adipose tissue in mice and humans
Source: Cell Death Dis. 2019 Jun 13;10(6):468. doi: 10.1038/s41419-019-1706-y (PMC6565685; doi:10.1038/s41419-019-1706-y)
Supplement: Supplementary file 1 — Supplemental data revised [file 41419_2019_1706_MOESM1_ESM.doc]

Supplemental Data

Berberine Promotes the Recruitment and Activation of Brown Adipose Tissue in mice and Humans

Lingyan Wu^1,2*^, Mingfeng Xia^3,5*^, Yanan Duan^1*^, Lina Zhang^1^, Haowen Jiang^1^, Xiaobei Hu^1^, Hongmei Yan^3,5^, Yiqiu Zhang^4^, Yushen Gu^4^, Hongcheng Shi^4^, Jia Li^1$^, Xin Gao^3,5$^ and Jingya Li^1$^

^1^State Key Laboratory of Drug Research, Shanghai Institute of Materia Medica, Chinese Academy of Sciences, Shanghai, P. R. China

^2^University of Chinese Academy of Sciences, Beijing, P.R. China

^3^Department of Endocrinology and Metabolism and ^4^Department of Nuclear Medicine, Zhongshan Hospital, Fudan University, Shanghai, P. R. China

^5^Fudan Institute for Metabolic Diseases, Shanghai, P. R. China

*These authors contributed equally to this work.

^$^Corresponding authors.


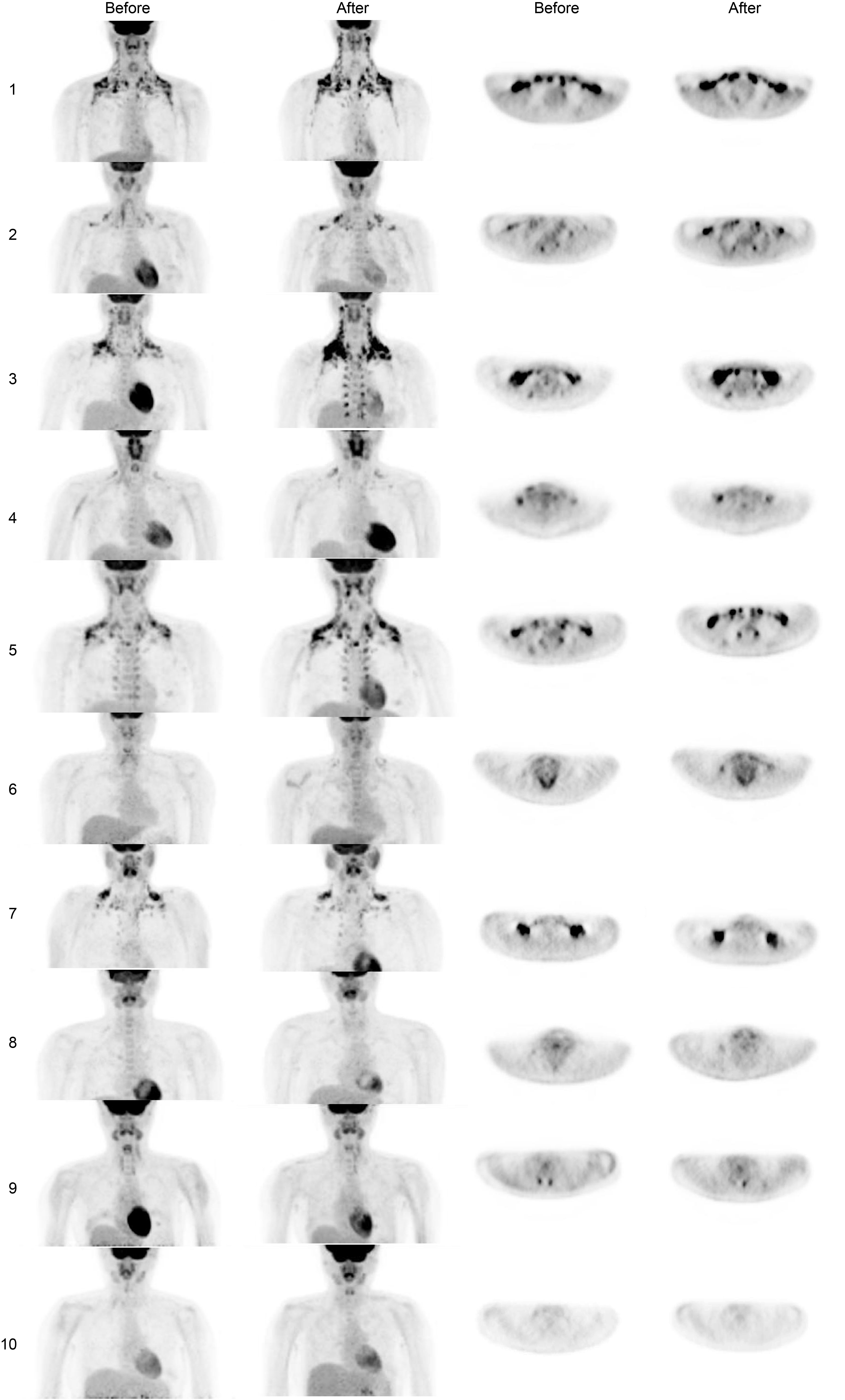


**Supplemental** **Figure 1. ^18^F-FDG PET/CT images indicating cold activated glucose uptake before and after BBR intervention in ten subjects.** Subject #9 and #10 had no detectable BAT neither before nor after BBR intervention.


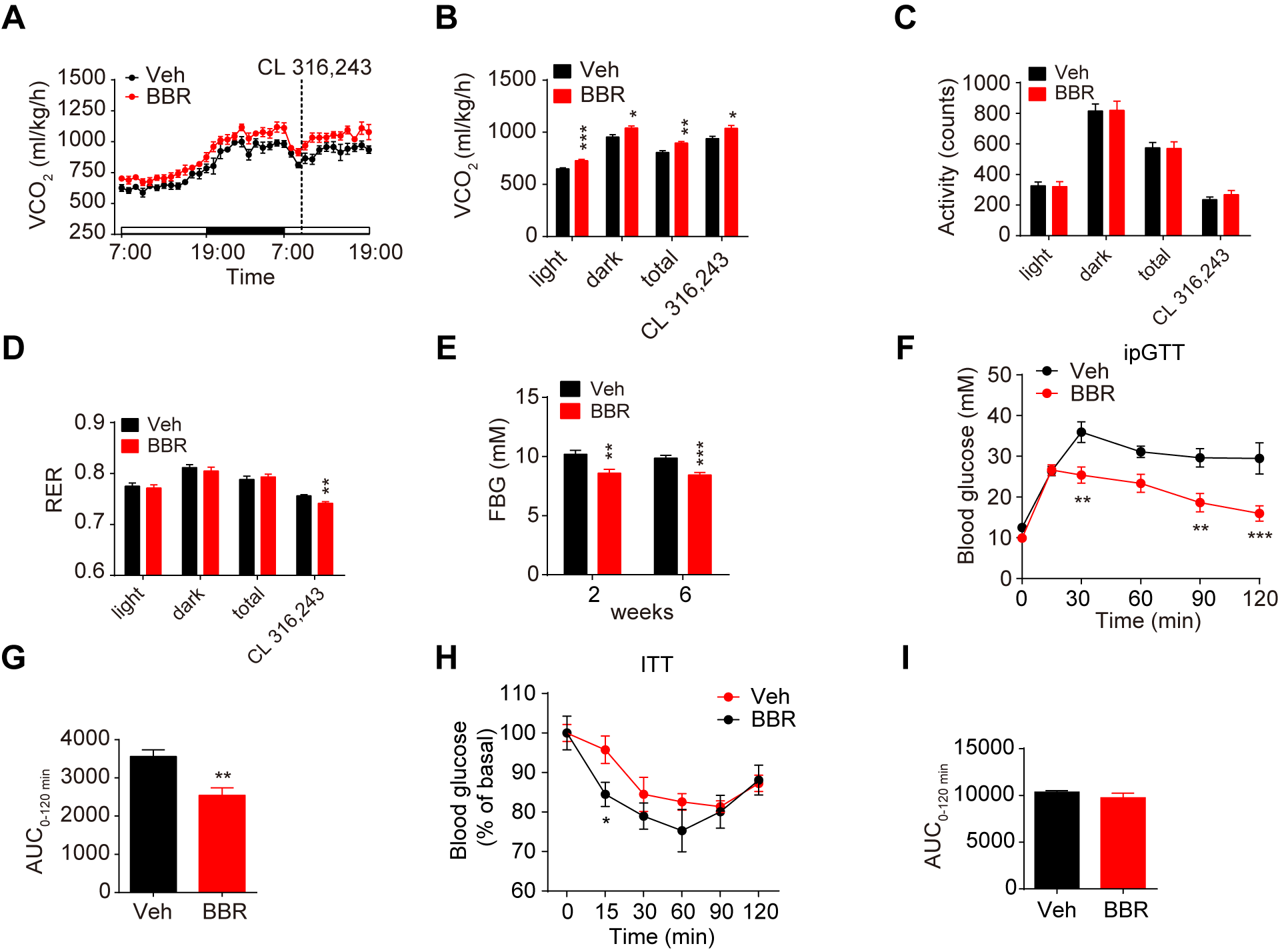


**Supplemental Figure 2.** **BBR increases energy expenditure and improves glucose tolerance in DIO mice.** **(A-D)** Metabolic analysis was performed in the 4^th^ week of treatment: CO_2_ production (A) and average CO_2_ production (B), average RER (C) and locomotor activity (D) during indicated period under basal and CL 316,243-stimulated condition. n = 7-8. **(E)** Fasting blood glucose (FBG) level of DIO mice in the 2^nd^ and 6^th^ week of treatment. n = 7-10. **(F-G)** Intraperitoneal glucose tolerance test (ipGTT) was conducted in the 4^th^ week of treatment (F), two-way ANOVA; The AUC during indicated time was calculated (G), two-tailed Student’s t test. n = 9-10. **(H-I)** Insulin tolerance test (ITT) in the 5^th^ week of the treatment (H), two-way ANOVA; the AUC during indicated time was calculated (I), two-tailed Student’s t test. n = 9-10. *P < 0.05, **P < 0.01, ***P < 0.001 compared with vehicle. Data are expressed as mean ± SEM.


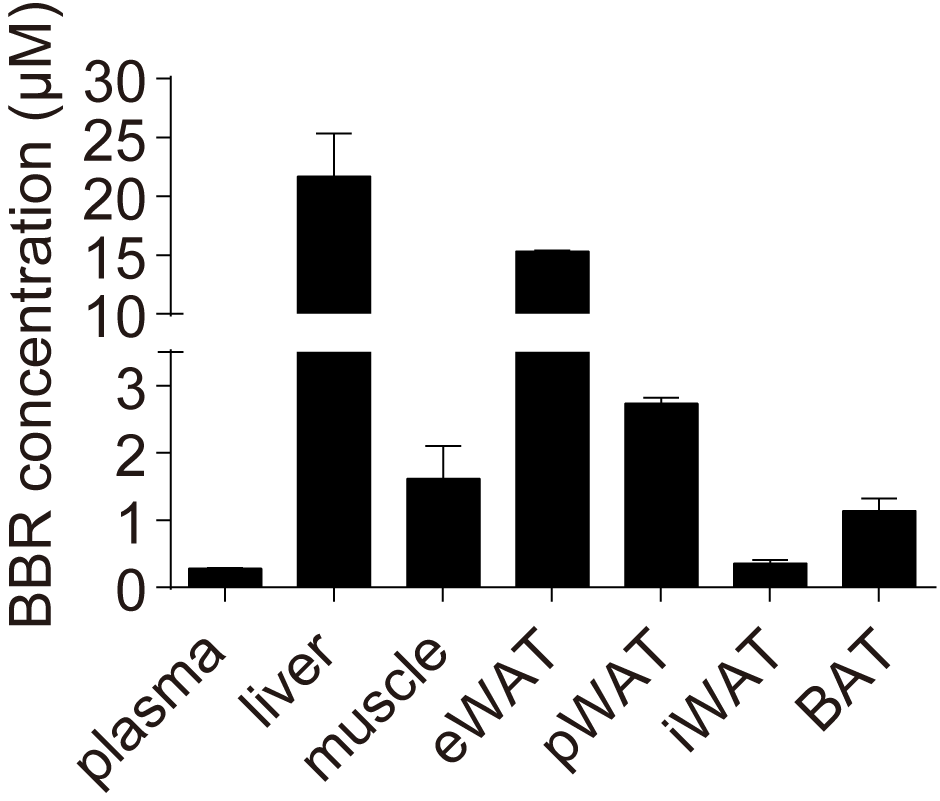


**Supplemental Figure 3. The tissue distribution of BBR in DIO mice.** Tissue distribution of BBR at 1 h after a bolus i.p. injection of BBR (5mg/kg) in DIO mice. The plasma and tissue samples were collected and the BBR concentration in different tissues were determined by liquid chromatography–mass spectrometry/mass spectrometry (LC-MS/MS). n = 3. Data are expressed as mean ± SEM.


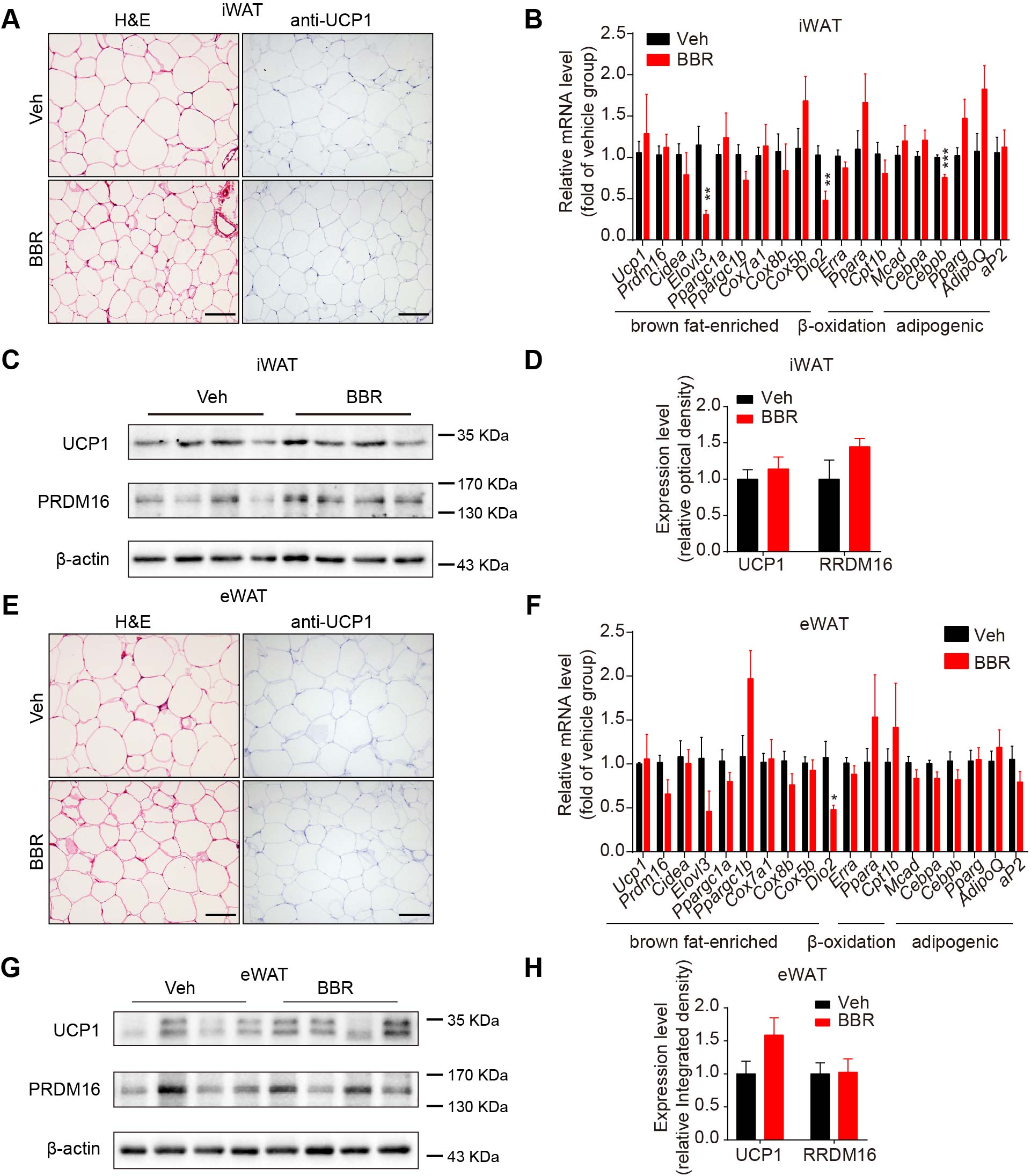


**Supplemental Figure 4. Chronic BBR treatment has no effect on the browning of inguinal or epididymal WAT in DIO mice. (A)** Representative H&E staining and UCP1 IHC of iWAT in DIO mice treated with vehicle or BBR for 6 weeks. n = 9-10. **(B)** Relative mRNA levels of indicated genes in inguinal WAT (iWAT) of DIO mice treated with vehicle or BBR for 6 weeks. n = 6-7. **(C-D)** Representative western bolt images of indicated proteins in iWAT of DIO mice after 6-weeks treatment (C). Relative optical density of UCP1/β-actin and PRDM16/β-actin were determined (D). n = 4. **(E)** Representative H&E staining and UCP1 IHC images of eWAT in DIO mice after 6-weeks of treatment. n = 8-10. **(F)** Relative mRNA levels of indicated genes in epididymal WAT (eWAT) of DIO mice after 6 weeks of treatment. n = 6-7. **(G-H)** Representative western bolt of indicated proteins in eWAT of DIO mice after 6-week treatment (G). Relative optical density of UCP1/β-actin and PRDM16/β-actin were determined (H). n = 4. *P < 0.05, **P < 0.01, ***P < 0.001 compared with vehicle by two-tailed Student’s t test. Data are expressed as mean ± SEM. Scale bar 100 μm.


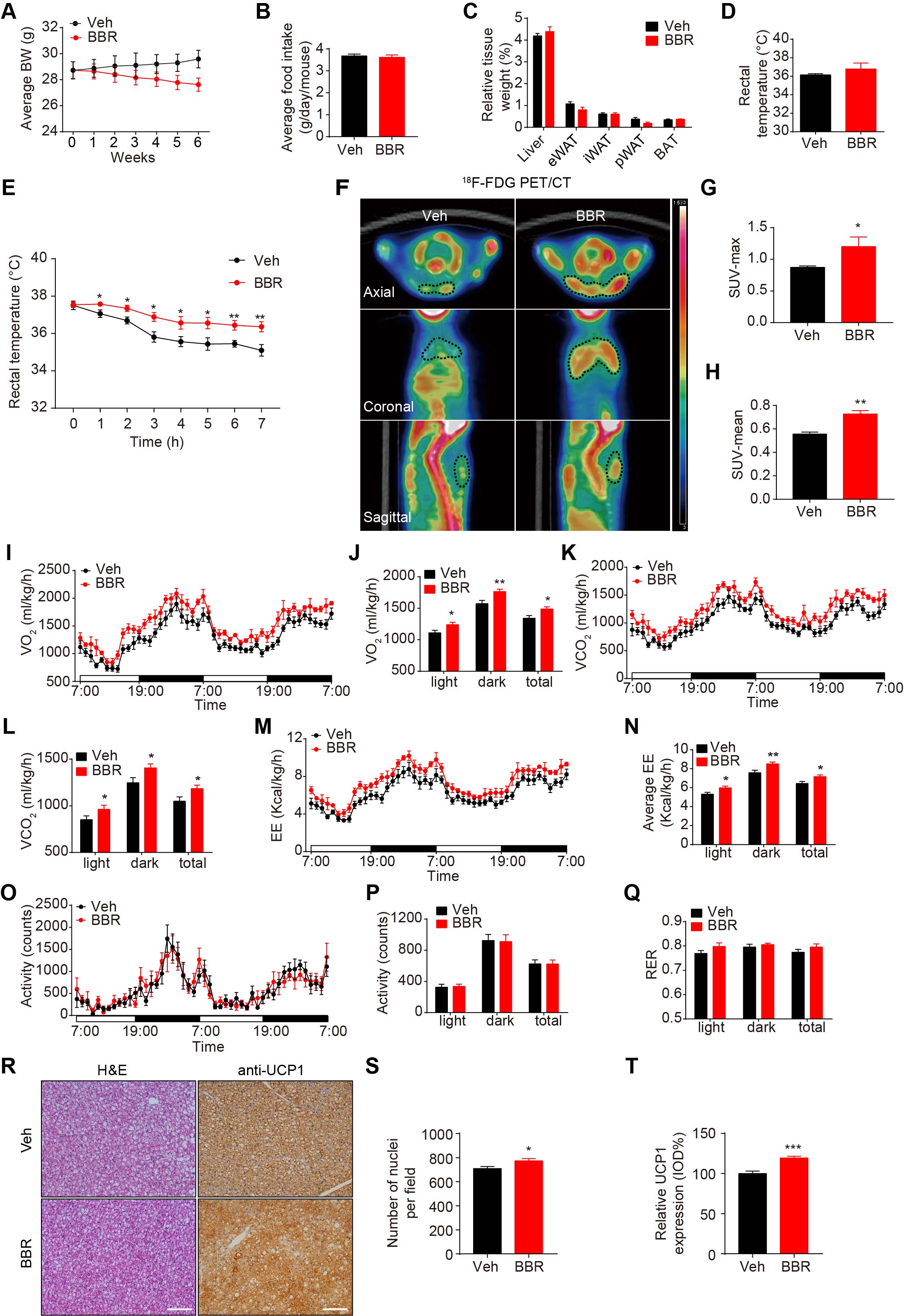


(Legend on next page)

**Supplemental Figure 5. Chronic BBR treatment enhances BAT thermogenesis and energy expenditure in chow-fed lean mice.** 14-week-old chow-fed lean mice (age-matched with DIO mice) were i.p. treated with vehicle or BBR (1.5mg/kg/day) for 6 weeks. Metabolic analysis was conducted in the 5^th^ week of the treatment. **(A-B)** Average body weight change (A) and average food intake (B). Two-way ANOVA in (A). n = 9. **(C)** Relative tissue weight of liver, eWAT, iWAT, pWAT and BAT fat pads to body weight. n= 8-9. **(D)** Rectal temperature of lean mice in the 6^th^ week of treatment. n = 7-8. **(E)** The rectal temperature of lean mice treated with vehicle or BBR for 6-weeks housed in cold temperature (4 °C) for 7 hrs. n = 9-10. **(F-G)** Representative axial, coronal and sagittal ^18^F-FDG PET/CT images of lean mice. Dashed lines mark the interscapular BAT region (F); Maximal SUV (G); Mean SUV (H). n = 4. **(I-Q)** O_2_ consumption change (I), average O_2_ consumption (J), CO_2_ production change (K), average CO_2_ production (L), EE change (M), average EE (N), locomotor activity change (O), average locomotor activity (P) and average RER (Q) of lean mice during two complete 12 h light-dark cycles. n = 7-9. **(R)** Representative H&E staining and UCP1 IHC images of BAT in lean mice. n = 6-8. Scale bar 100 μm. **(S-T)** Quantification of nuclei number of brown adipocytes (S) and relative UCP1 expression depicted by IOD % (T) in the sections shown in (R). 4-8 slides/mouse. *P < 0.05, **P < 0.01, ***P < 0.001 compared with vehicle by two-tailed Student’s t test. Data are expressed as mean ± SEM.


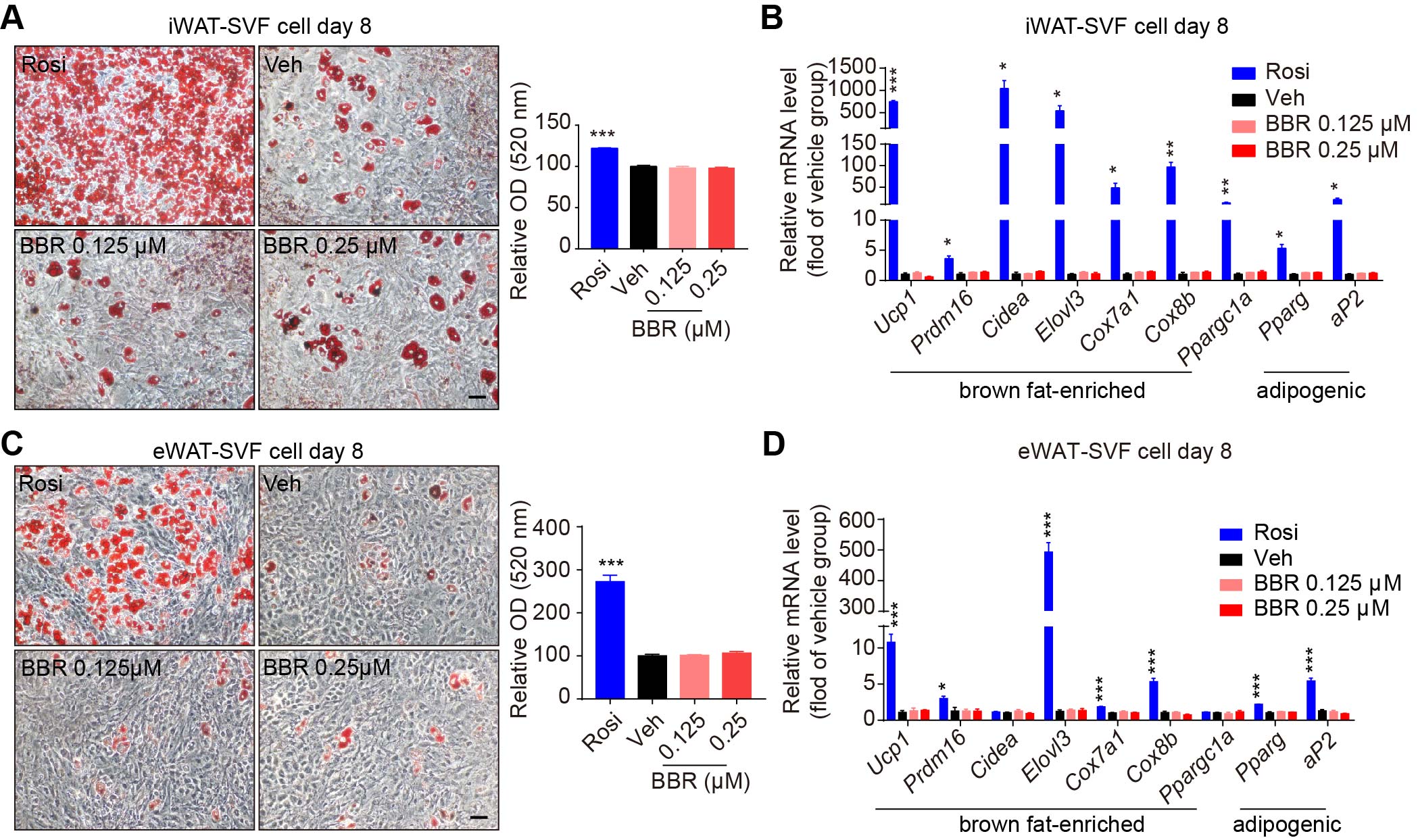


**Supplemental Figure 6. BBR has no effect on beige adipogenesis of iWAT-SVF or eWAT-SVF cells. (A)** Representative oil red O staining of SVF cells isolated from iWAT after inducing beige adipogenesis in the presence or absence of BBR for 8 days (left). Relative OD measurement at 520 nm were determined (right). n = 4. **(B)** Relative mRNA levels of indicated genes in iWAT-SVF cells at day 8 of differentiation. n = 3. **(C)** Representative oil red O staining of SVF cells isolated from eWAT after inducing beige adipogenesis in the presence or absence of BBR for 8 days (left). Relative OD measurement at 520 nm were determined (right). n = 3. **(D)** Relative mRNA levels of indicated genes in eWAT-SVF cells at day 8 of differentiation. n = 4. Rosiglitazone (Rosi) as a positive control. Two-tailed Student’s t test, *P < 0.05, **P < 0.01, ***P < 0.001, Veh versus Rosi; One-way ANOVA, Veh versus BBR. Data are expressed as mean ± SEM. Scale bar 100 μm.


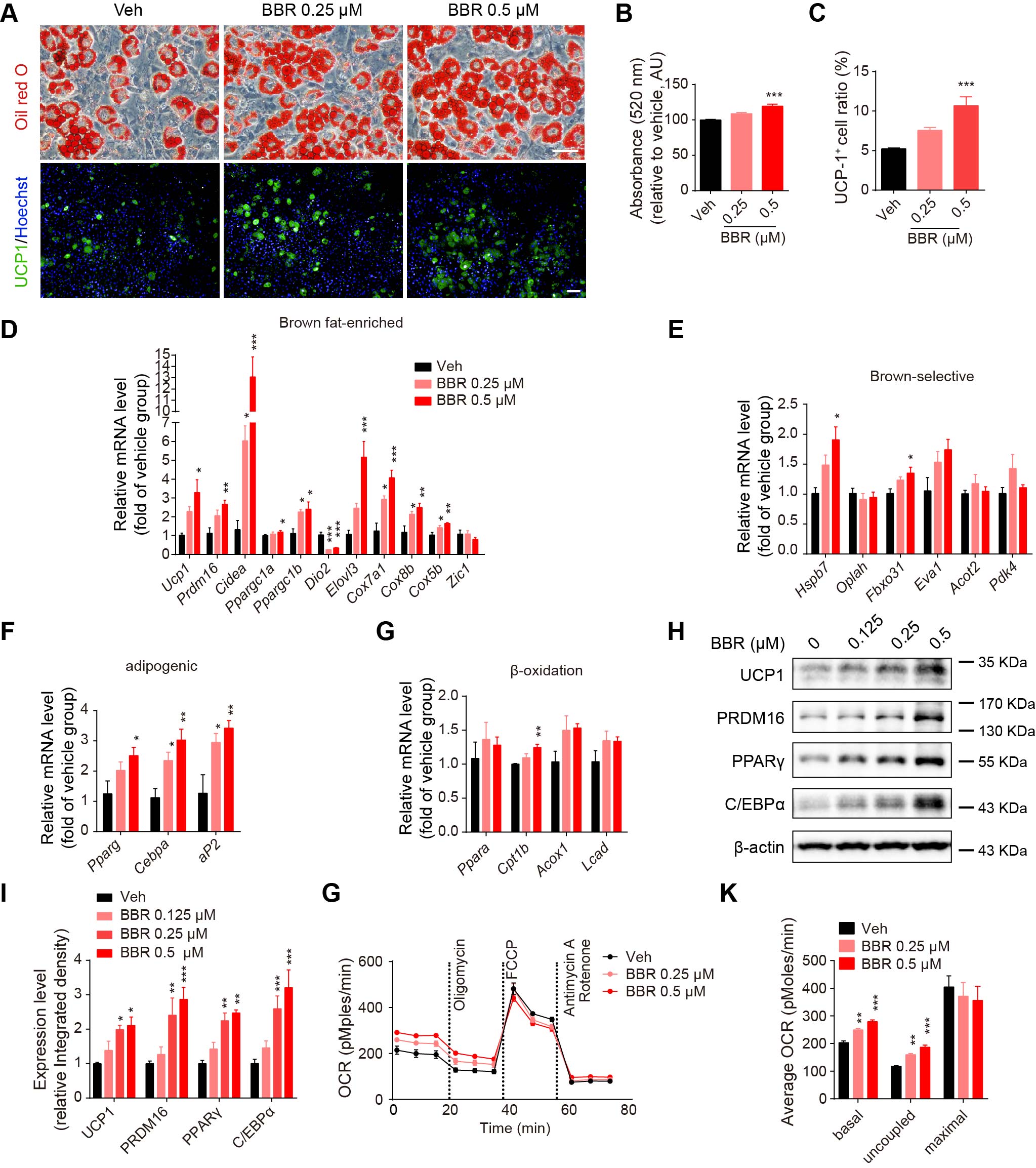


**Supplemental Figure 7. BBR promoted brown adipogenesis in mesenchymal progenitor cells. (A-C)** Representative oil red O staining and immunofluorescence staining of UCP1 (green) and Hoechst (blue) of C3H10-T1/2 cells after brown adipogenesis induction in the presence or absence of BBR for 8 days (A); Relative OD measurement at 520 nm (B); UCP1 positive cell ratio (C). **(D-G)** Relative mRNA levels of indicated genes in C3H10-T1/2 cells at day 8 of differentiation. **(H-I)** Representative western bolt analysis (H) and relative integrated density (I) of indicated protein levels in C3H10-T1/2 cells on day 8 of differentiation. **(G-K)** Real-time change OCR (G) and average OCR (K) of C3H10-T1/2 cells under basal, uncoupled and maximal conditions on day 8 of differentiation. Data are expressed as mean ± SEM. *P < 0.05, **P < 0.01, ***P < 0.001 compared with vehicle by Student’s t test or One-way ANOVA followed by Dunnett’s multiple comparisons for comparison of two or more groups


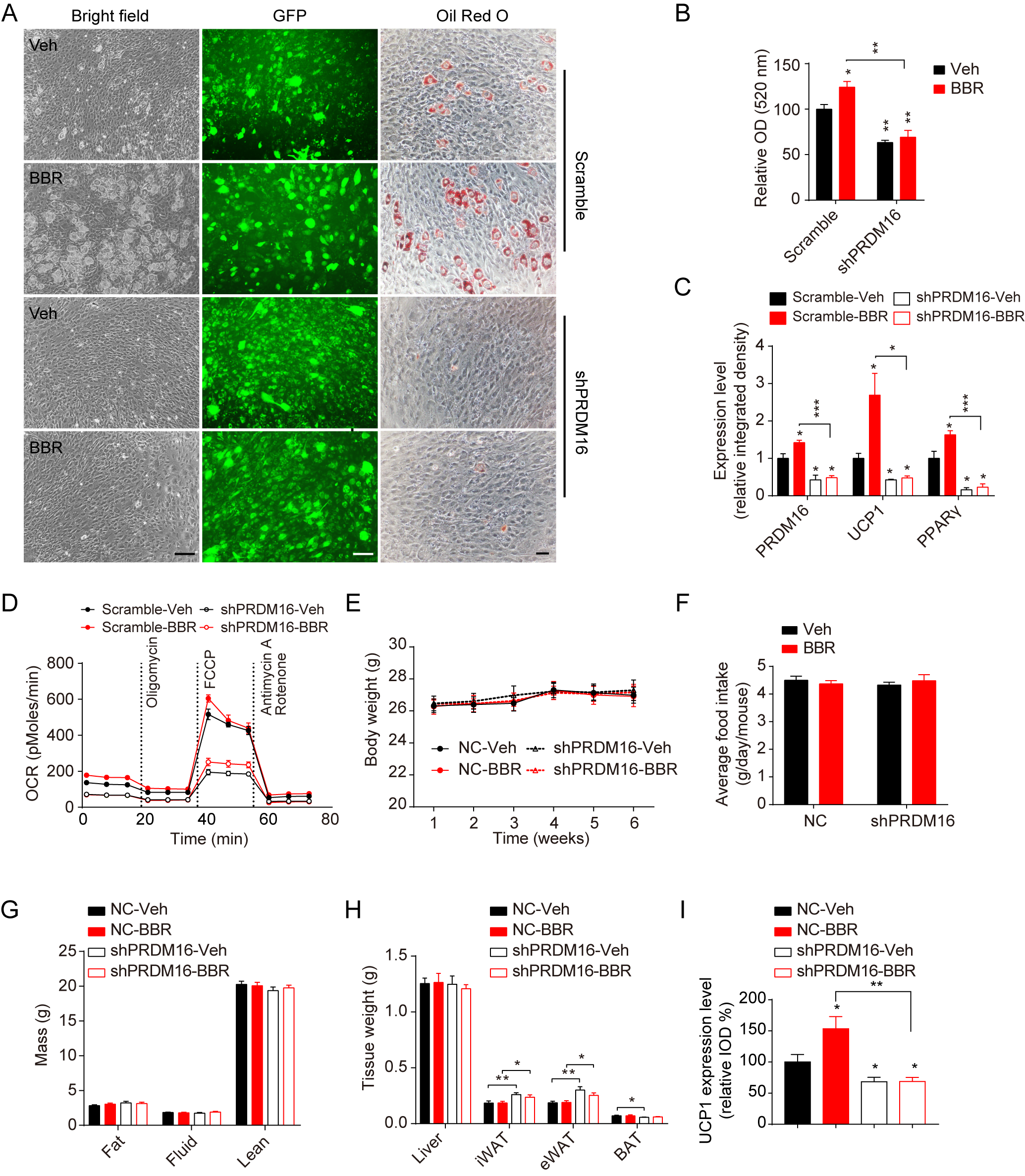


**Supplemental Figure 8. PRDM16 is required for BBR-promoted brown adipogenesis. (A-B)** Representative bright field, immunofluorescence staining of GFP and oil red O staining of scramble or shPRDM16 stable cells after brown adipogenesis induction in the presence of vehicle or BBR for 8 days (A). Quantification of oil red O staining (B). Scale bar 100 μm. n = 4. **(C)** Relative expression levels of indicate proteins were determined by densitometric qualification of the immunoblots shown in Figure 4F. n = 3. **(D)** Average OCR in scramble and PRDM16 stable cells measured under basal, uncoupled, maximal and non-mitochondrial conditions on day 8 of differentiation. n = 4. **(E-H)** Body weight (E) and food intake (F) of BAT-specific PRDM16 knockdown mice and control NC mice during the treatment. The absolute fat mass, fluid mass and lean mass of BAT-specific PRDM16 knockdown mice and control NC mice were measured by using NMR spectrum analyzer after 6-weeks of treatment (G). The absolute weight of liver, iWAT, eWAT, pWAT and BAT of BAT-specific PRDM16 knockdown mice and NC mice after 6-weeks treatment (H). n = 9-10. **(I)** Relative integrated density of indicated proteins in BAT of BAT-specific PRDM16 knockdown mice and NC mice after 6-weeks treatment. n = 4. In A-D, BBR 0.5 μM. *P < 0.05, **P < 0.01, ***P < 0.001 compared with vehicle by two-tailed Student’s t test. Data are expressed as mean ± SEM.


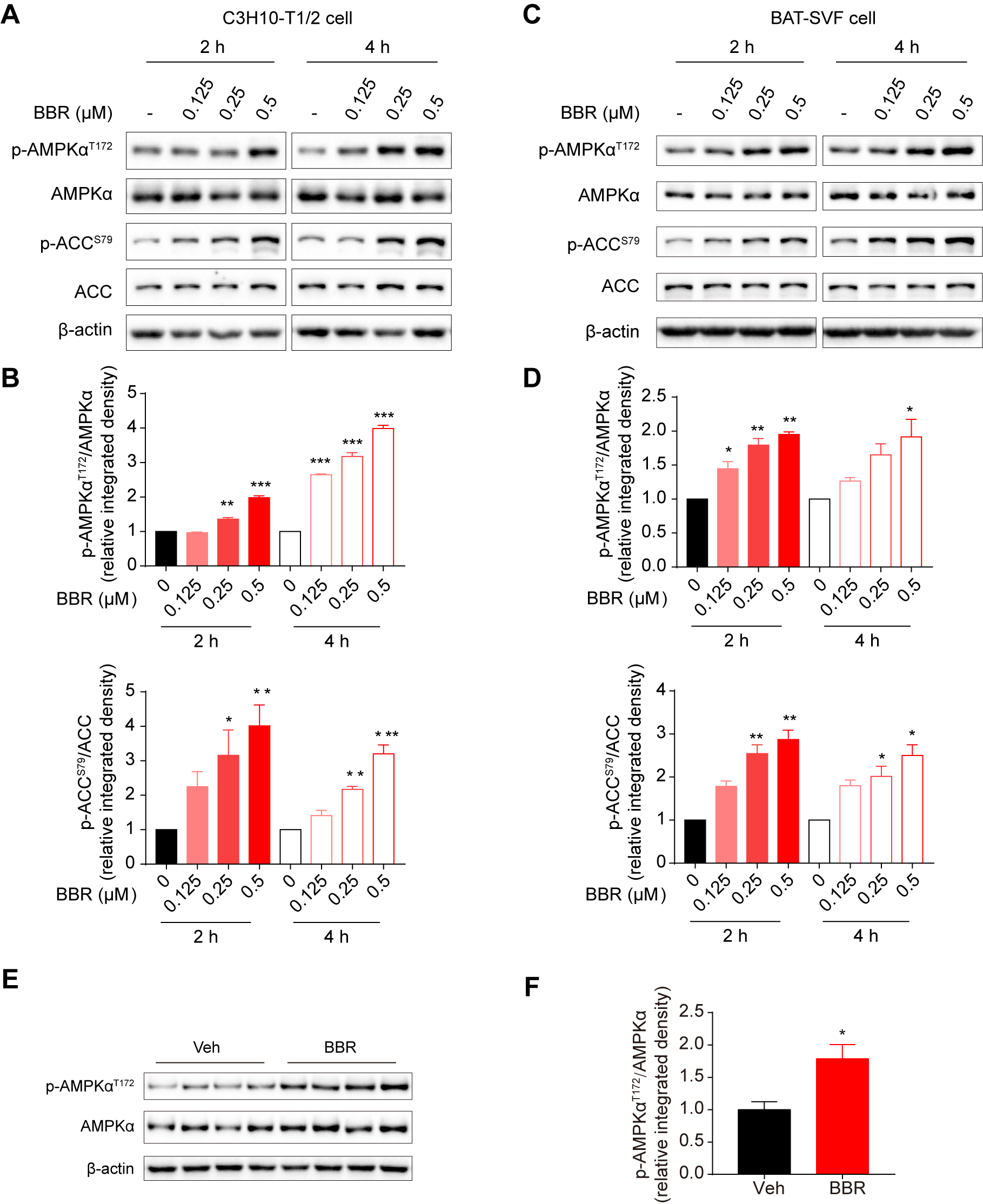


**Supplemental Figure 9. BBR activates AMPK signaling *in vitro* and *in vivo*. (A-B)** C3H10-T1/2 cells were induced to differentiating into brown adipocytes and treated with indicated compounds for 2 h or 4h. Western blot analysis of the indicated proteins were shown (A) and relative optical density of p-AMPK^T172^/AMPK and p-ACC^S79^/ACC were determined (B). β-actin was used as a loading control. n = 3. **(C-D)** BAT-SVF cells were induced to differentiating into brown adipocytes and treated with indicated compounds for 2 h or 4h. Western blot analysis of the indicated proteins were shown (C) and relative optical density of p-AMPK^T172^/AMPK and p-ACC^S79^/ACC were determined (D). n = 3. **(E-F)** Representative Western blot analysis of indicated proteins (E) and relative integrated density (F) of p-AMPK^T172^/AMPK in BAT of lean mice after 6-weeks treatment. n = 4. Data are expressed as mean ± SEM. *P < 0.05, **P < 0.01, ***P < 0.001 compared with vehicle by Student’s t test or One-way ANOVA followed by Dunnett’s multiple comparisons for comparison of two or more groups.


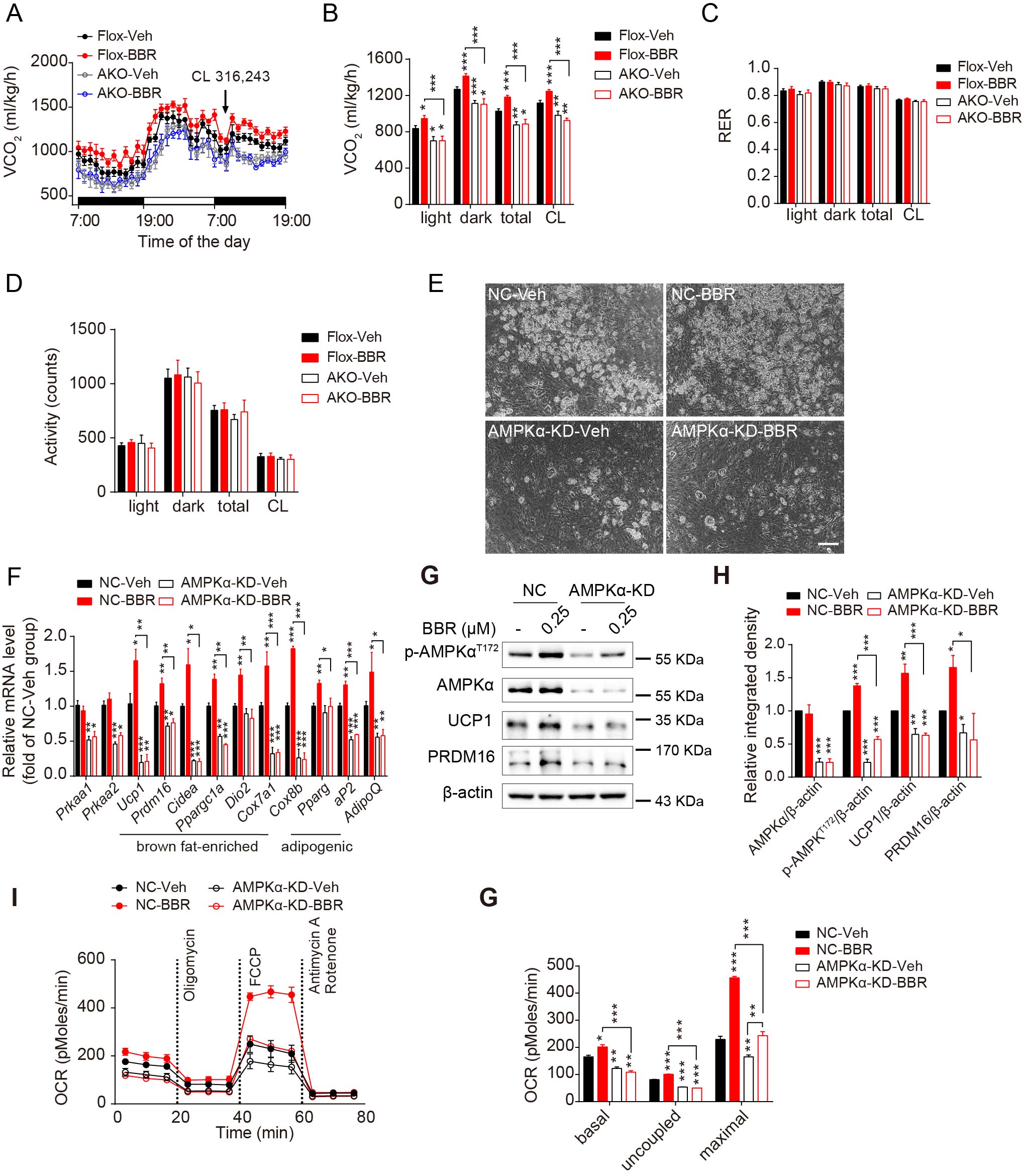


**Supplemental Figure 10. AMPK knockdown blunted BBR-promoted brown adipocyte differentiation. (A-D)** The CO_2_ production change (A) and average CO_2_ production (B), RER (C) and activity (D) of AKO and floxed mice under basal condition and after single injection with CL 316,243 were measured by using an indirect calorimeter after 4-weeks treatment. n = 8. **(E-G)** BAT-SVF cells were infected with scramble-cas9 (NC) or sgAMPKα1-cas9/sg AMPKα2-cas9 lentivirus (AMPKα knockdown (KD)) prior to differentiation induction and concurrently treatment with vehicle or BBR through 8 days of differentiation. Representative bright field images (E); The mRNA levels of the indicated genes (F); Representative western blot images (G) and the relative integrated density (H) of the indicated proteins; OCR change (I) and average basal, uncoupled and maximal OCR (G). Data are expressed as mean ± SEM. *P < 0.05, **P < 0.01, ***P < 0.001 compared with Flox-Veh or NC-Veh by Student’s t test.


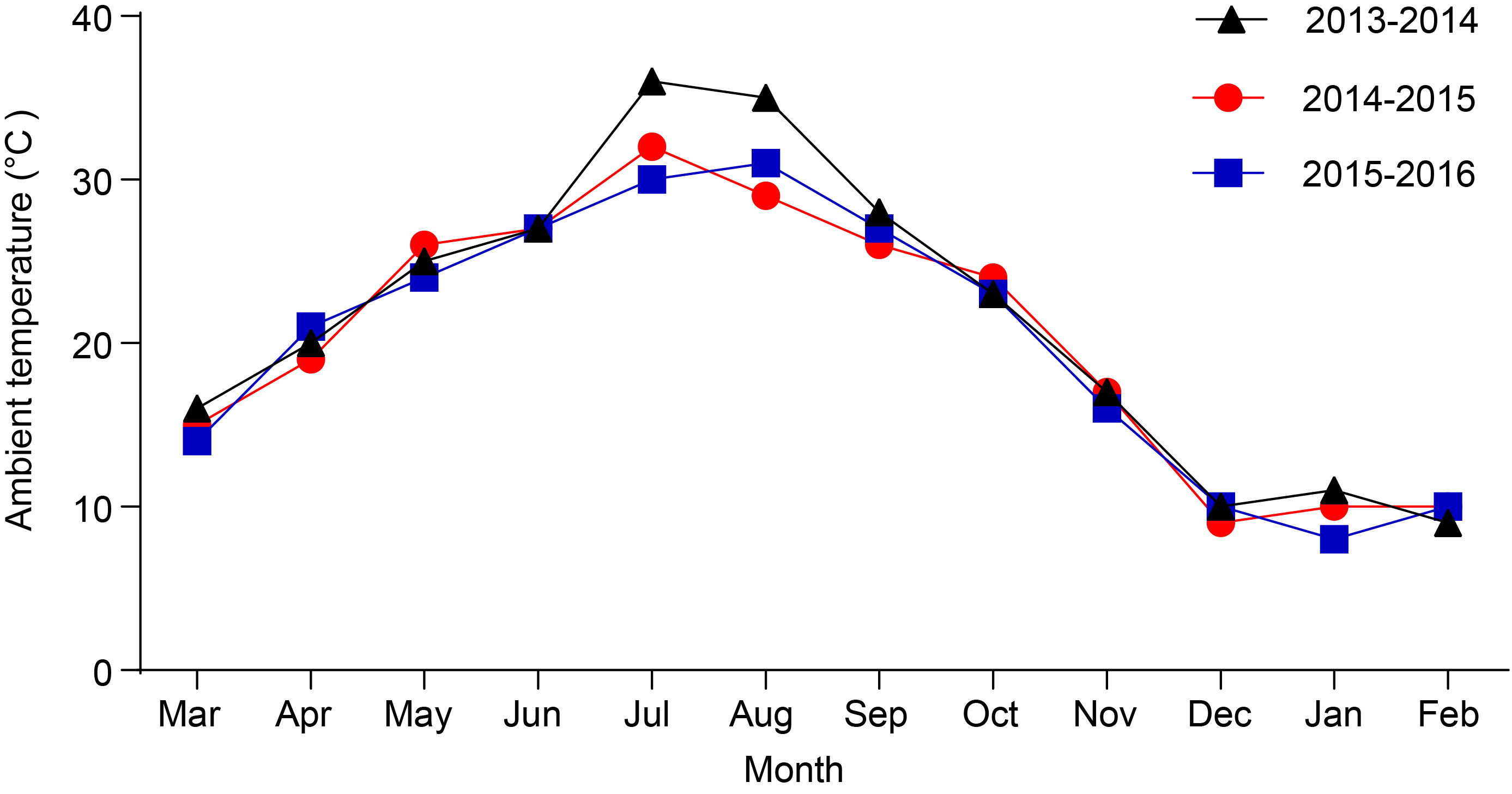


**Supplemental Figure 11.** **The ambient temperature from March 2013 to February 2016 in Shanghai, China.**

| **Supplemental Table 1.** Comparison of metabolic parameters of NAFLD patients before and after BBR intervention | | | |
| --- | --- | --- | --- |
| Parameters | Before (n = 10) | After (n = 10) | P value^A^ |
| TG (mmol/L) | 0.92 ± 0.24 | 0.98 ± 0.34 | 0.62 |
| TC (mmol/L) | 4.8 ± 1.7 | 4.4 ± 1.4 | 0.12 |
| HDL-C (mmol/L) | 1.3 ± 0.3 | 1.2 ± 0.1 | 0.23 |
| LDL-C (mmol/L) | 3.0 ± 1.6 | 2.6 ± 1.4 | 0.13 |
| NEFA (mEq/L) | 0.50 ± 0.16 | 0.53 ± 0.21 | 0.46 |
| Leptin (ng/mL) | 3.54 (1.68 - 15.3) | 5.19 (2.87 - 17.3) | 0.20 |
| Glucagon (pg/mL) | 68.2 ± 40.3 | 67.5 ± 31.8 | 0.94 |
| ALT (U/L) | 17.6 ±11.1 | 20.6 ±12.1 | 0.46 |
| AST (U/L) | 18.2 ± 3.7 | 17.8 ±4.4 | 0.78 |
| LFC (%) | 14.7 (9.7 - 29.7) | 13.3 (10.3 - 25.8) | 0.83 |
| Lactic acid (mg/dL) | 16.9 ± 7.2 | 19.6 ± 6.4 | 0.29 |
| free T4 (pmol/L) | 17.8 ± 2.9 | 17.7 ± 2.6 | 0.69 |
| TSH (μU/mL) | 2.5 ± 1.2 | 2.3 ± 0.9 | 0.66 |
| ACTH (pg/mL) | 36.8 ± 3.4 | 37.8 ± 5.5 | 0.45 |
| Epinephrine (pg/mL) | 163.5 (64.9 - 340.0) | 169.5 (116.7 - 609.0) | 0.17 |
| Irisin (ng/mL) | 13.6 ± 4.2 | 12.9 ± 2.3 | 0.50 |
| FGF21 (pg/mL) | 113.5 ± 62.4 | 106.2 ± 32.0 | 0.84 |
| ^A^ Paired two-tailed t test. TC: total cholesterol, NEFA: nonesterified fatty acid, ALT: alanine transaminase, AST: aspartate transaminase, LFC%: liver fat content, TSH: thyroid stimulating hormone, ACTH: adrenocorticotropic hormone, FGF21: fibroblast growth factor 21. | | | |

| **Supplemental Table 2.** Plasma parameters in DIO and lean mice | | | | |
| --- | --- | --- | --- | --- |
| Parameters | DIO-Veh | DIO-BBR | lean-Veh | lean-BBR |
| TC (mM) | 8.20 ± 0.85 | 8.08 ± 0.60 | 3.31 ± 0.2 | 4.73 ± 1.28 |
| LDL (mM) | 1.73 ± 0.11 | 1.41 ± 0.08* | 0.67 ± 0.06 | 0.71 ± 0.09 |
| HDL (mM) | 0.50 ± 0.05 | 0.59 ± 0.06 | 0.43 ± 0.03 | 0.47 ± 0.05 |
| LDL/HDL | 3.71 ± 0.34 | 2.58 ± 0.17* | 1.57 ± 0.07 | 1.60 ± 0.29 |
| TG (mM) | 0.40 ± 0.03 | 0.37 ± 0.02 | 0.49 ± 0.051 | 0.43 ±0 .05 |
| Insulin (ng/mL) | 3.946 ± 0.60 | 2.12 ± 0.22** | 1.49 ± 0.02 | 1.56 ± 0.06 |
| Leptin (ng/mL) | 49.19 ± 4.65 | 18.27 ± 5.28*** | 1.12 ± 0.22 | 1.46 ± 0.32 |
| NEFA (mEq/L) | 0.61 ± 0.12 | 0.44 ± 0.04 | 0.83 ± 0.08 | 0.66 ± 0.04 |
| Irisin (ng/mL) | 113.56 ± 3.23 | 109.33 ± 3.13 | 108.10 ± 6.88 | 107.66 ± 5.90 |
| Values are expressed as mean ± SEM; Two-tailed t test was used, *P < 0.05; **P < 0.01; ***P < 0.001 compared with the corresponding vehicle group. In DIO mice, n = 10. In lean mice, n = 6-8. | | | | |

| **Supplemental Table 3.** Primer sequences used in quantitative RT-PCR assays | | |
| --- | --- | --- |
| Gene | Forward 5’-3’ | Reverse 5’-3’ |
| *Ucp1* | ACTGCCACACCTCCAGTCATT | CTTTGCCTCACTCAGGATTGG |
| *Prdm16* | CAGCACGGTGAAGCCATTC | GCGTGCATCCGCTTGTG |
| *Cidea* | TGCTCTTCTGTATCGCCCAGT | GCCGTGTTAAGGAATCTGCTG |
| *Ppargc1a* | TATGGAGTGACATAGAGTGTGCT | CCACTTCAATCCACCCAGAAAG |
| *Ppargc1b* | GTCCCTGGCTGACATTCACT | GCACGGATCTCATGGTCTCT |
| *Dio2* | AATTATGCCTCGGAGAAGACCG | GGCAGTTGCCTAGTGAAAGGT |
| *Elovl3* | TTCTCACGCGGGTTAAAAATGG | GAGCAACAGATAGACGACCAC |
| *Cox7a1* | CTCTTCCAGGCCGACAATGA | GCCCAGCCCAAGCAGTATAA |
| *Cox8b* | GAACCATGAAGCCAACGACT | GCGAAGTTCACAGTGGTTCC |
| *Cox5b* | GCTGCATCTGTGAAGAGGACAAC | CAGCTTGTAATGGGTTCCACAGT |
| *Ppara* | GCGTACGGCAATGGCTTTAT | GAACGGCTTCCTCAGGTTCTT |
| *Cpt1b* | AGGCACTTCTCAGCATGGTC | ACGGACACAGATAGCCCAGA |
| *Acox1* | TAACTTCCTCACTCGAAGCCA | AGTTCCATGACCCATCTCTGTC |
| *Lcad* | TCACCAACCGTGAAGCTCGA | CCAAAAAGAGGCTAATGCCATG |
| *Mcad* | AGCTGCTAGTGGAGCACCAAG | TCGCCATTTCTGCGAGC |
| *Pparg* | TTCCGAAGAACCATCCGATTG | TGGCATTGTGAGACATCCCCAC |
| *Cebpa* | AGTACCGGGTACGGCGGGAAC | GCGTGTCCAGTTCACGGCTCA |
| *Cebpb* | TCGGGACTTGATGCAATCC | AAACATCAACAACCCCGC |
| *Cebpd* | GCTTTGTGGTTGCTGTTGAA | ATCGACTTCAGCGCCTACA |
| *aP2* | ACACCGAGATTTCCTTCAAACTG | CCATCTAGGGTTATGATGCTCTTCA |
| *AdipoQ* | TGACGACACCAAAAGGGCTC | CACAAGTTCCCTTGGGTGGA |
| *Hspb7* | GAGCATGTTTTCAGACGACTTTG | CCGAGGGTCTTGATGTTTCCTT |
| *Oplah* | CTTCACGCACGTCTCCTTGT | GCATCTGCACAGGCCGTAT |
| *Fbxo31* | AAACTGCTTCACCGATACAGAC | ACCACGACGTTCAGCAATCC |
| *Eva1* | CCACTTCTCCTGAGTTTACAGC | GCATTTTAACCGAACATCTGTCC |
| *Acot2* | ATGGTGGCCTCGTCTTTCG | GAGCGGCGGAGGTACAAAC |
| *Pdk4* | AGGGAGGTCGAGCTGTTCTC | GGAGTGTTCACTAAGCGGTCA |
| *Erra* | GCAGGGCAGTGGGAAGCTA | CCTCTTGAAGAAGGCTTTGCA |
| *Ntrk3* | TGGCTCACACTGATCTCTGG | GCCAGAGCCTTTACTGCATC |
| *Zic1* | CTGTTGTGGGAGACACGATG | CCTCTTCTCAGGGCTCACAG |
| *Th* | CCAAGGTTCATTGGACGGC | CTCTCCTCGAATACCACAGCC |
| *Myod1* | TGCTCTGATGGCATGATGGATT | AGATGCGCTCCACTATGCTG |
| *Shh* | AAAGCTGACCCCTTTAGCCTA | TTCGGAGTTTCTTGTGATCTTCC |
| *36b4* | GAAACTGCTGCCTCACATCCG | GCTGGCACAGTGACCTCACACG |
| *18S* | AGTCCCTGCCCTTTGTACACA | CGATCCGAGGGCCTCACT |
| *Npy* | ATGCTAGGTAACAAGCGAATGGGG | TGAAATCAGTGTCTCAGGGCTGGA |
| *Agrp* | CGGAGGTGCTAGATCCACAGA | AGGACTCGTGCAGCCTTACAC |
| *Pomc* | CATAGATGTGTGGAGCTGGTG | CATCTCCGTTGCCAGGAAACAC |
| *Mc4r* | TGATCTGTAGCTCCTTGCTCGCAT | TGATGTTATGGTACTGGAGCGCGT |
| *Mc3r* | TCCTGCTGCCTGTCTTCTGTTTCT | ATGAAGACCTGCTCACAGAACCCA |

| **Supplemental Table 4.** Primer sequences used in ChIP-qPCR and (h)MeDIP | | |  |
| --- | --- | --- | --- |
| Gene | Forward 5’-3’ | Reverse 5’-3’ | Ref. |
| *Ucp1* enhancer | TCTACAGCGTCACAGAGGGT | TGATTTCTGCTCTTCTGGCA | [^1^](#_ENREF_1) |
| *Ucp1* TSS | AGTCCCACTAGCAGCTCTTTG | GACCCGTTAAGCCCAGATT | [^1^](#_ENREF_1) |
| *Cidea* (13k) | GGCCACTTGAGGAGCCAACCA | TGGGCACTGGCCTTGTAGCTG | [^2^](#_ENREF_2) |
| *aP2* (PPRE) | GACAAAGGCAGAAATGCACA | AATGTCAGGCATCTGGGAAC | [^3^](#_ENREF_3) |
| *18S* | AGTCCCTGCCCTTTGTACACA | CGATCCGAGGGCCTCACT | [^2^](#_ENREF_2) |
| *Prdm16 (h)MeDIP A* | AGAGAGAAGTGAGGTGAAGACCGAGAA | ACACACTATCTTCATCTCCCTAGCATTGT | [^4^](#_ENREF_4) |
| *Prdm16 (h)MeDIP B* | GCATGTGCGAAGGTGTCCAAA | TGGATCGCATGGTGTCGGCT | [^4^](#_ENREF_4) |
| *Prdm16 (h)MeDIP C* | CCAAAGCTTGAAGGAGAGAGACGTAAA | TCGGTTCTTGGCCTCCAGAGAAG | [^4^](#_ENREF_4) |
| *IAP* (repeat) | CTCCATGTGCTCTGCCTTCC | CCCCGTCCCTTTTTTAGGAGA | [^5^](#_ENREF_5) |

**References**

1. Iida S, Chen W, Nakadai T, Ohkuma Y, Roeder RG. PRDM16 enhances nuclear receptor-dependent transcription of the brown fat-specific Ucp1 gene through interactions with Mediator subunit MED1. *Genes & development* 2015, **29**(3)**:** 308-321.

2. Harms MJ, Lim HW, Ho Y, Shapira SN, Ishibashi J, Rajakumari S*, et al.* PRDM16 binds MED1 and controls chromatin architecture to determine a brown fat transcriptional program. *Genes & development* 2015, **29**(3)**:** 298-307.

3. Rajakumari S, Wu J, Ishibashi J, Lim HW, Giang AH, Won KJ*, et al.* EBF2 Determines and Maintains Brown Adipocyte Identity. *Cell metabolism* 2013, **17**(4)**:** 562-574.

4. Yang Q, Liang X, Sun X, Zhang L, Fu X, Rogers CJ*, et al.* AMPK/alpha-Ketoglutarate Axis Dynamically Mediates DNA Demethylation in the Prdm16 Promoter and Brown Adipogenesis. *Cell Metab* 2016, **24**(4)**:** 542-554.

5. Mohn F, Weber M, Schubeler D, Roloff TC. Methylated DNA immunoprecipitation (MeDIP). *Methods Mol Biol* 2009, **507:** 55-64.
