# Supplementary figures and images for: Berberine promotes the recruitment and activation of brown adipose tissue in mice and humans
Source: Cell Death Dis. 2019 Jun 13;10(6):468. doi: 10.1038/s41419-019-1706-y (PMC6565685; doi:10.1038/s41419-019-1706-y)

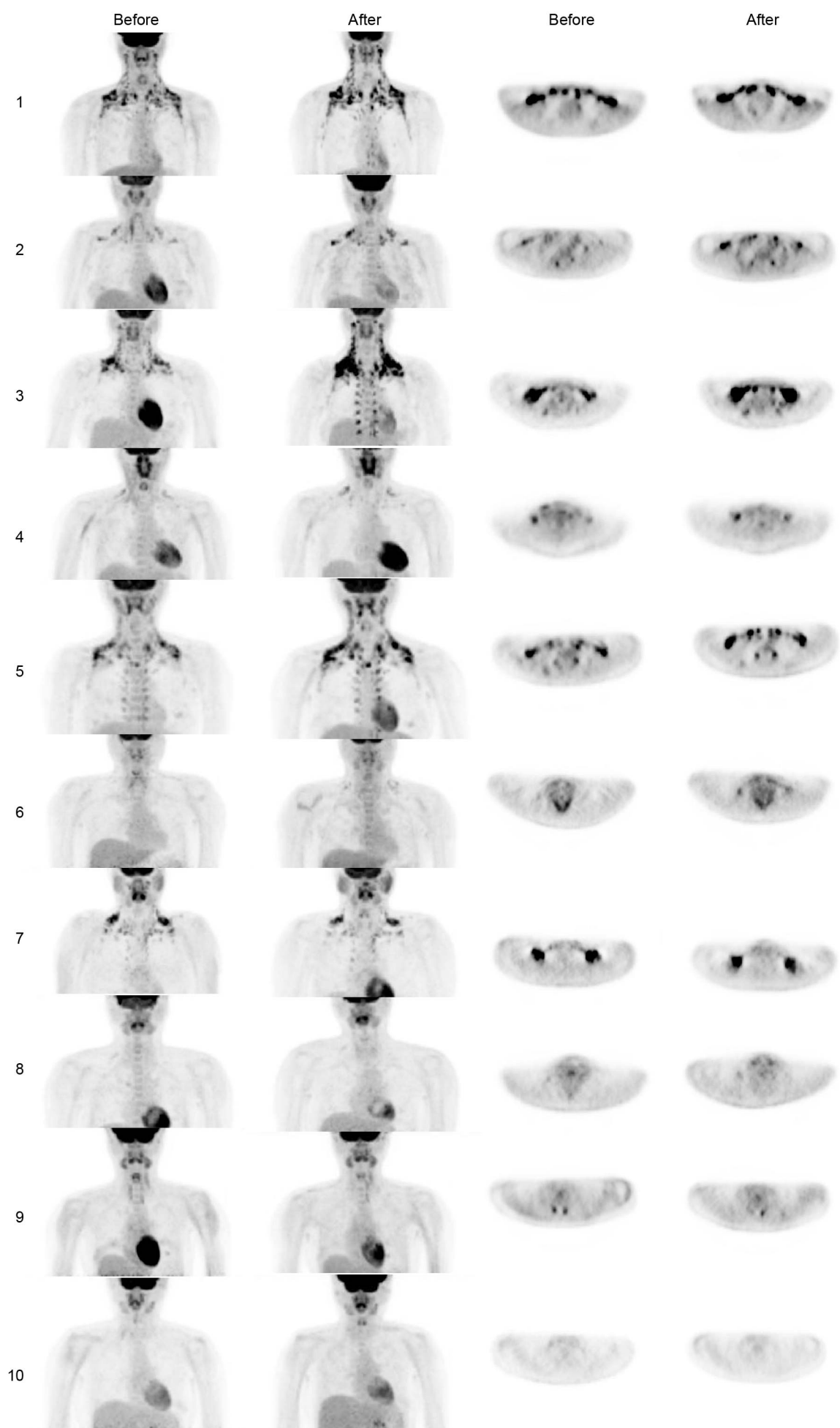

**A**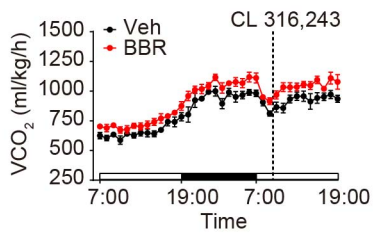**B**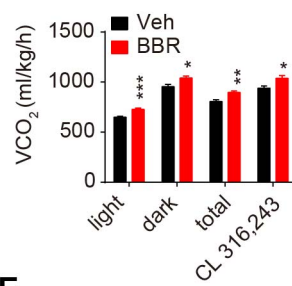**C**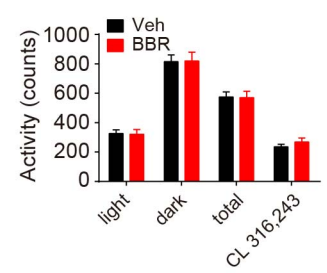**D**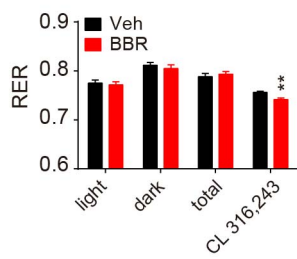**E**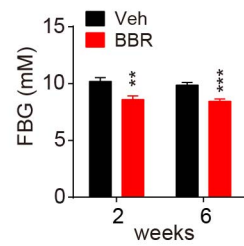**F**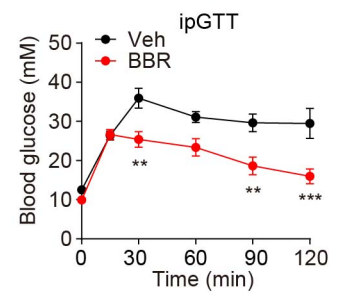**G**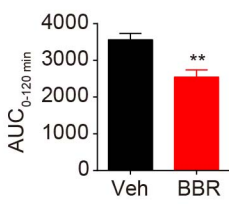**H**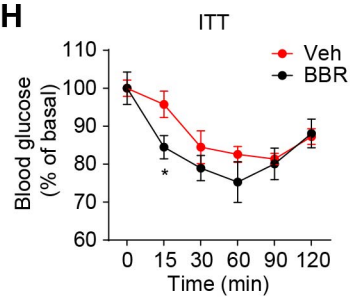**I**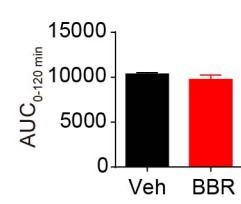

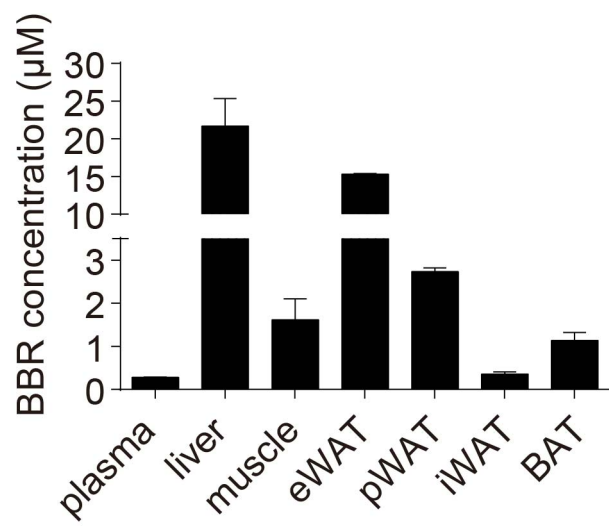

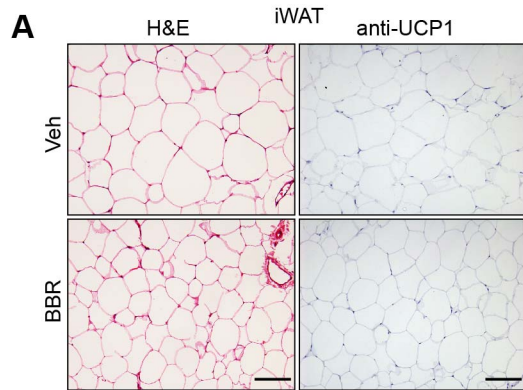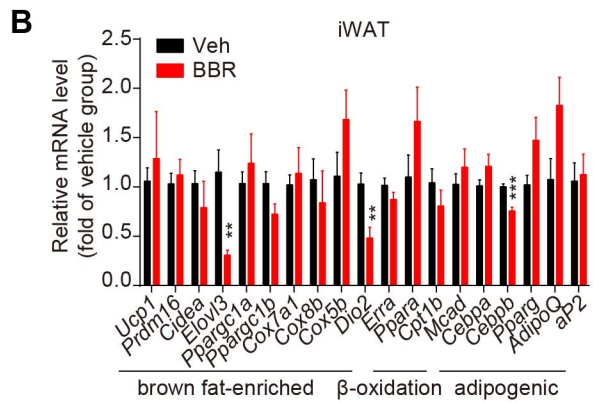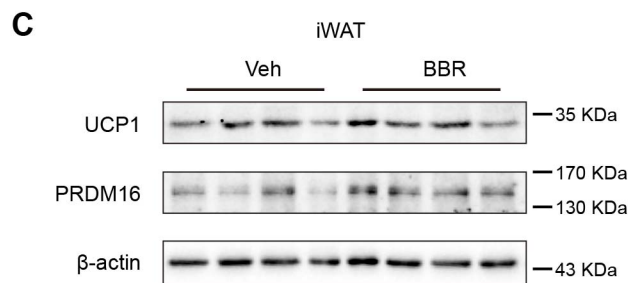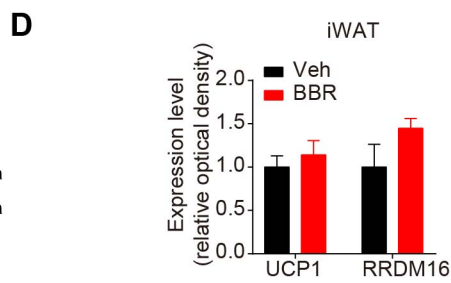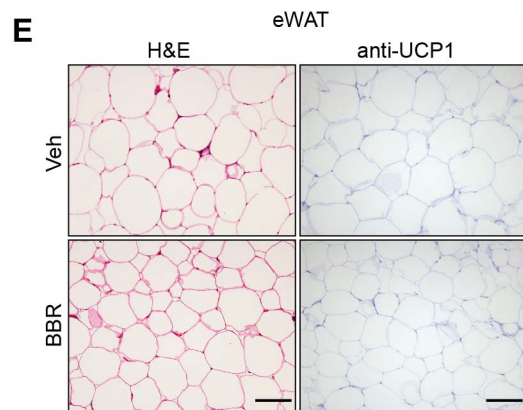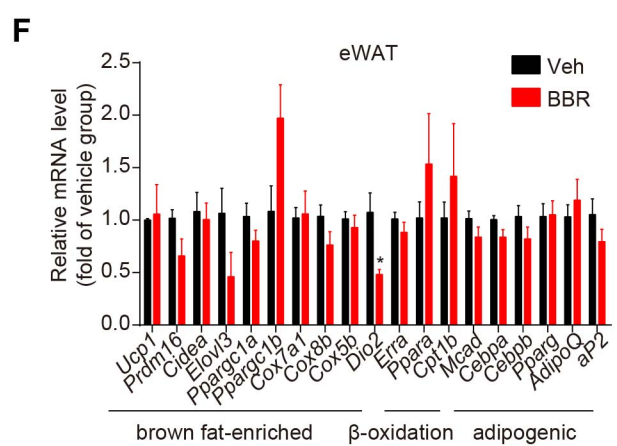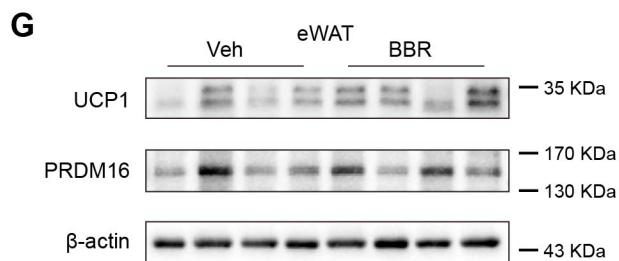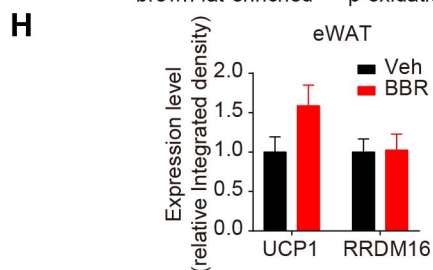

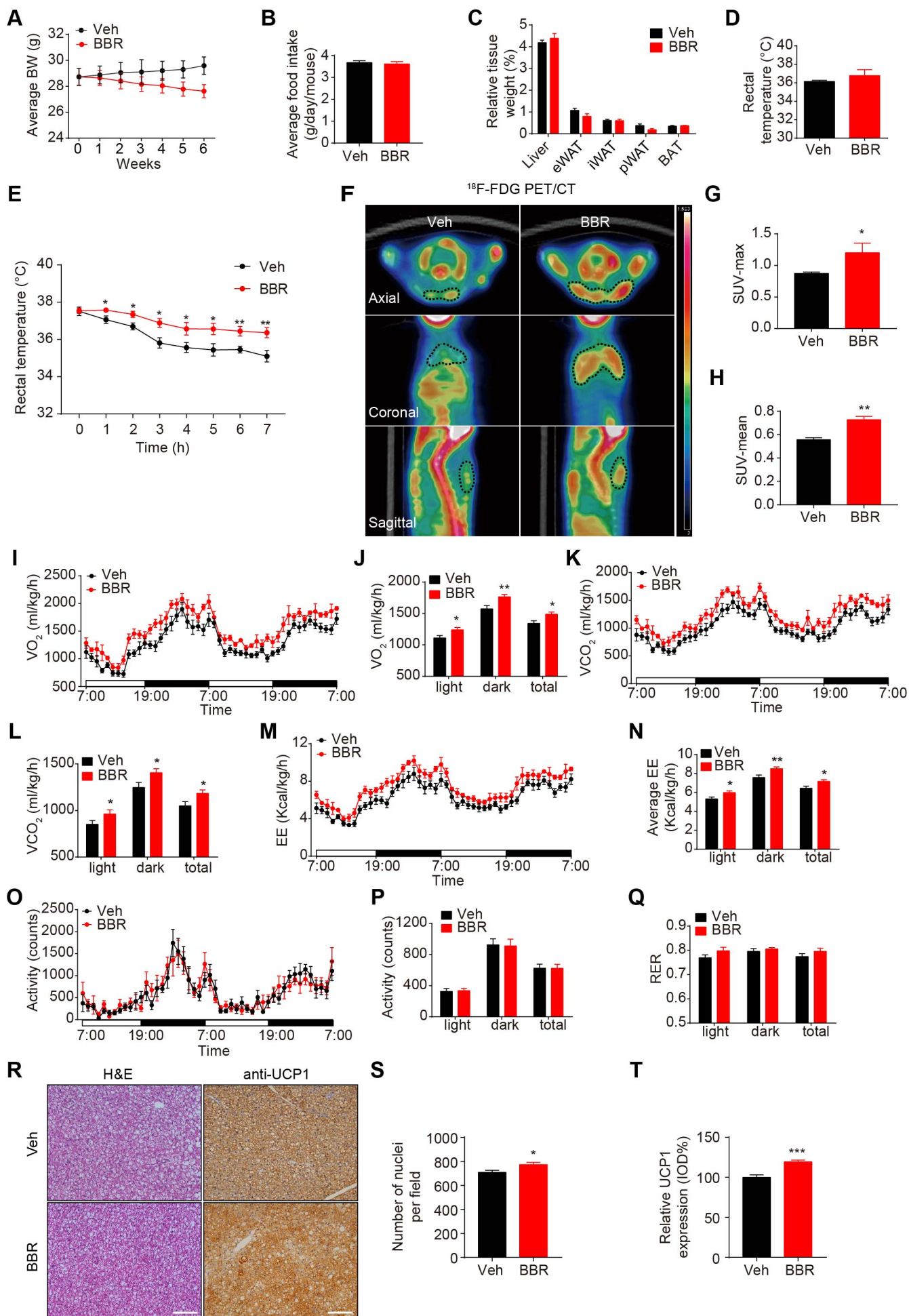

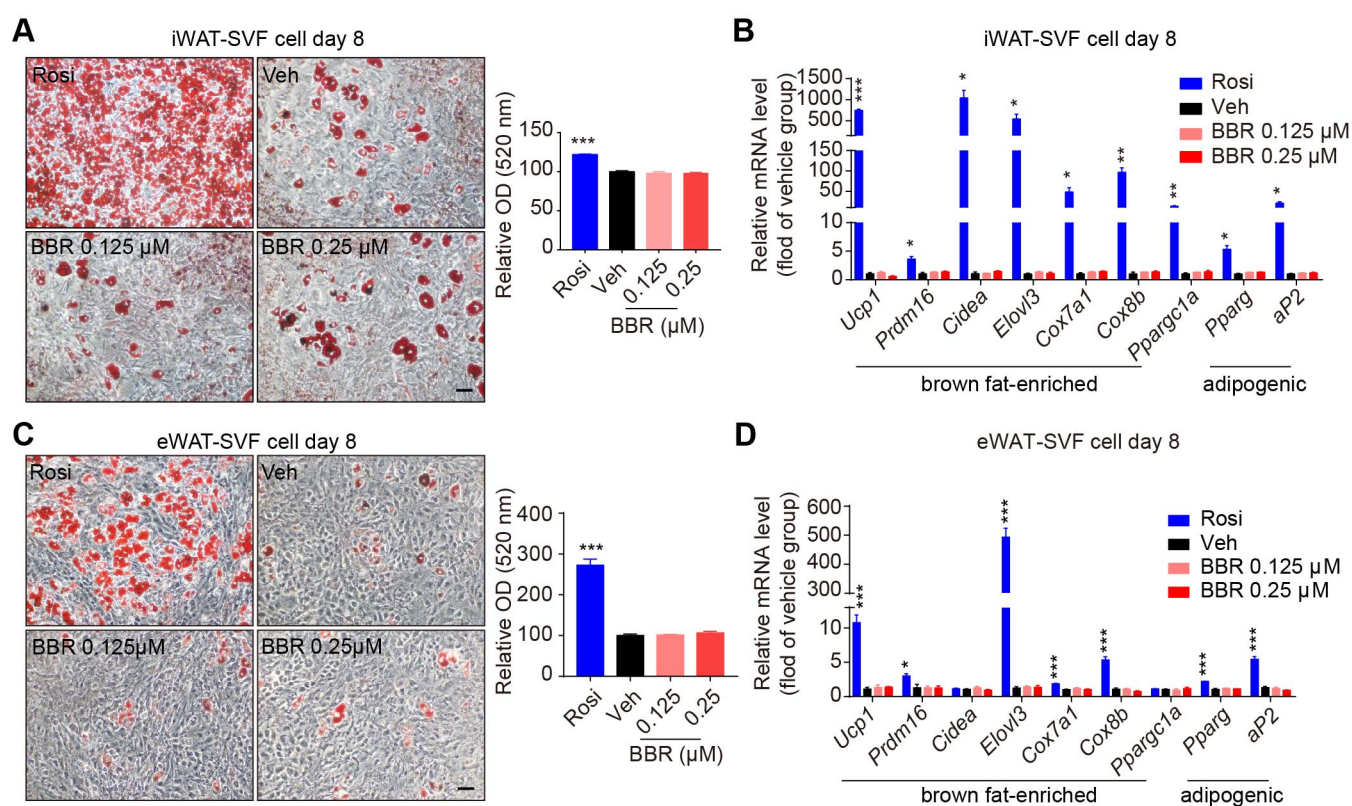

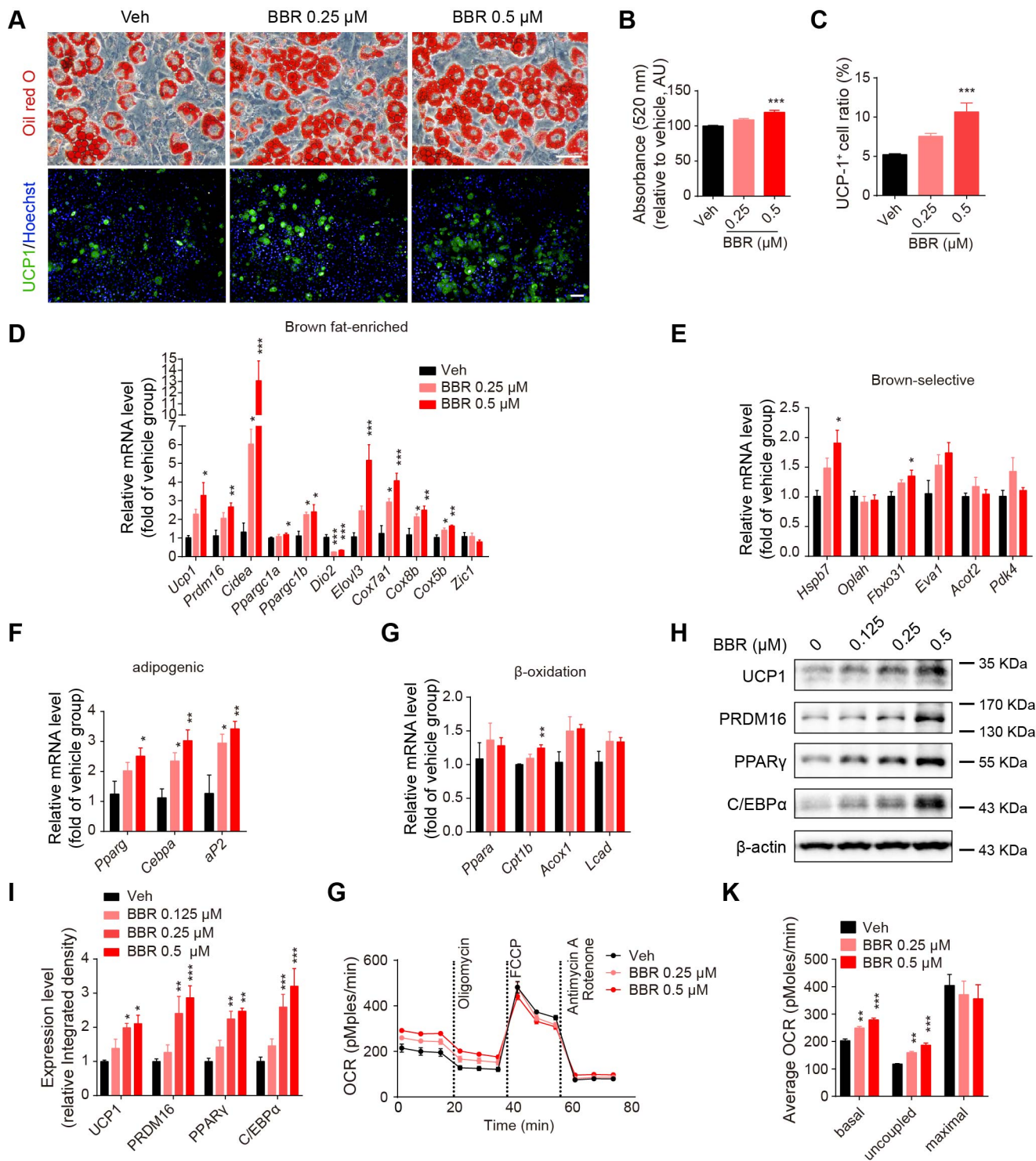

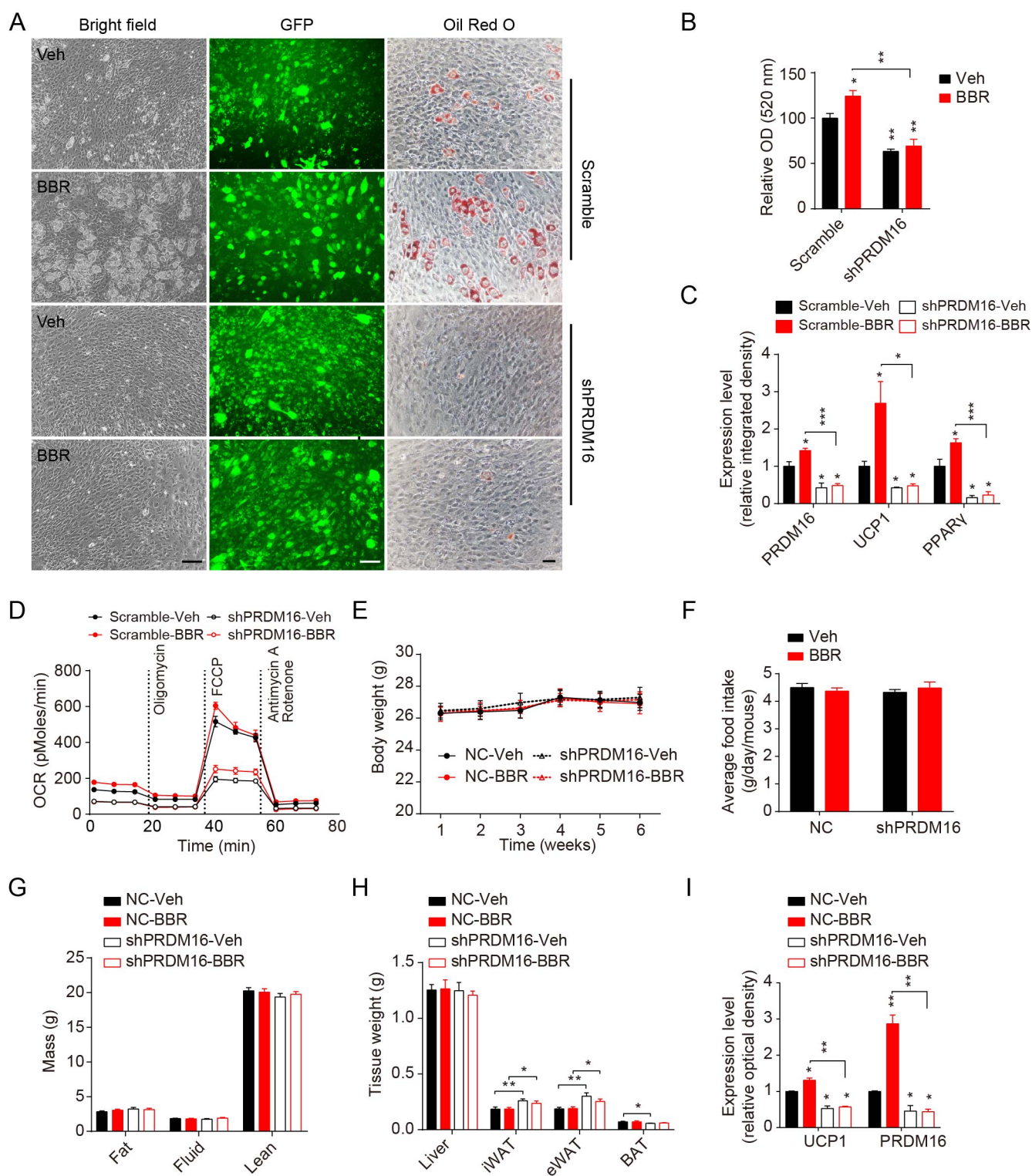

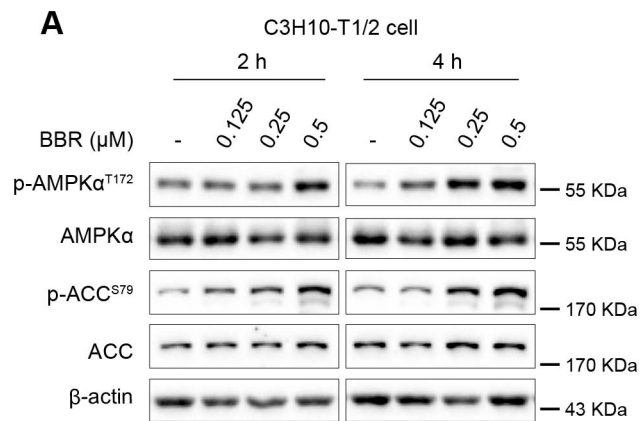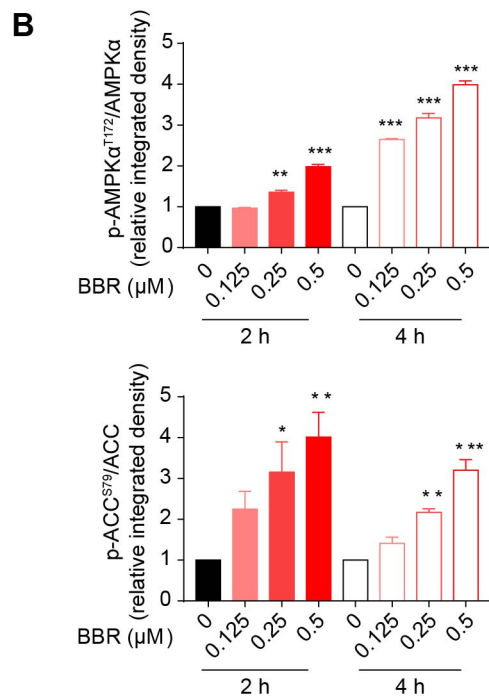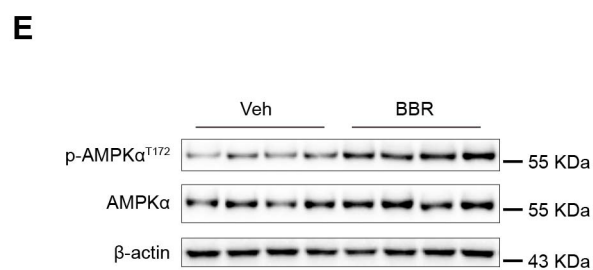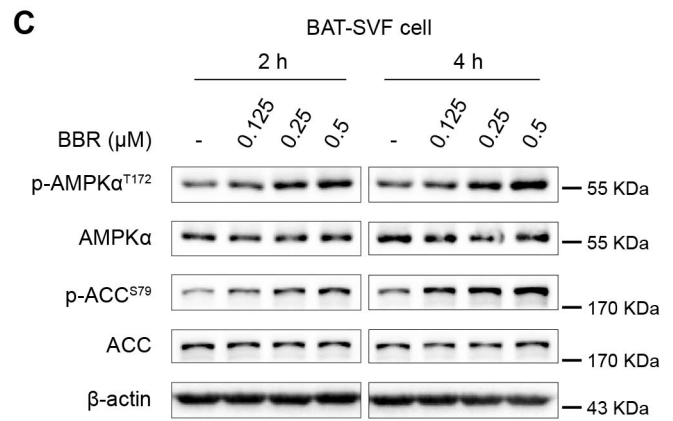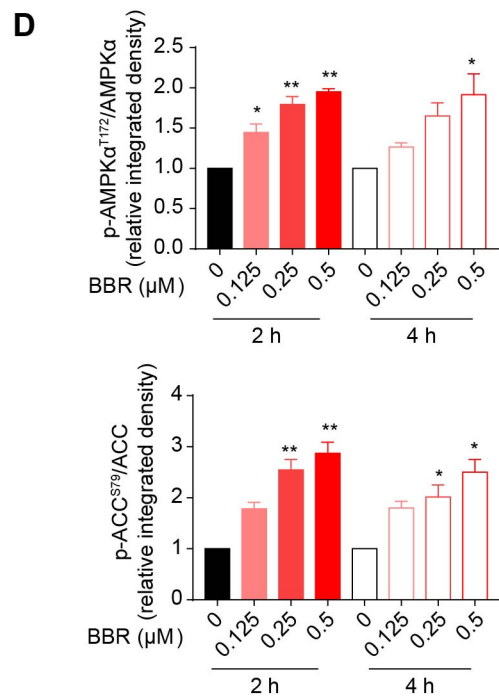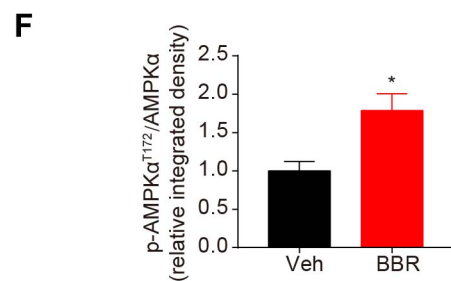

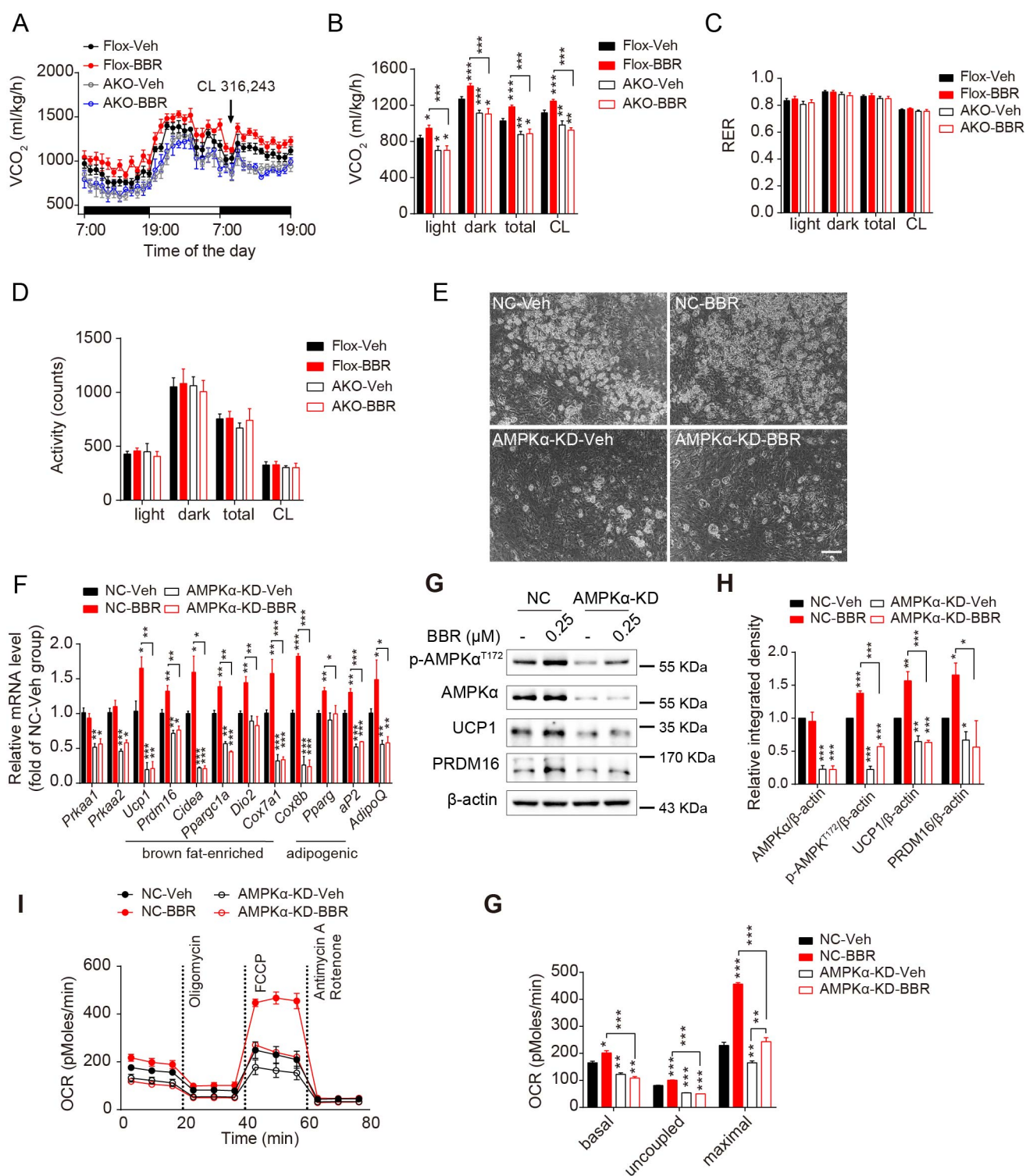

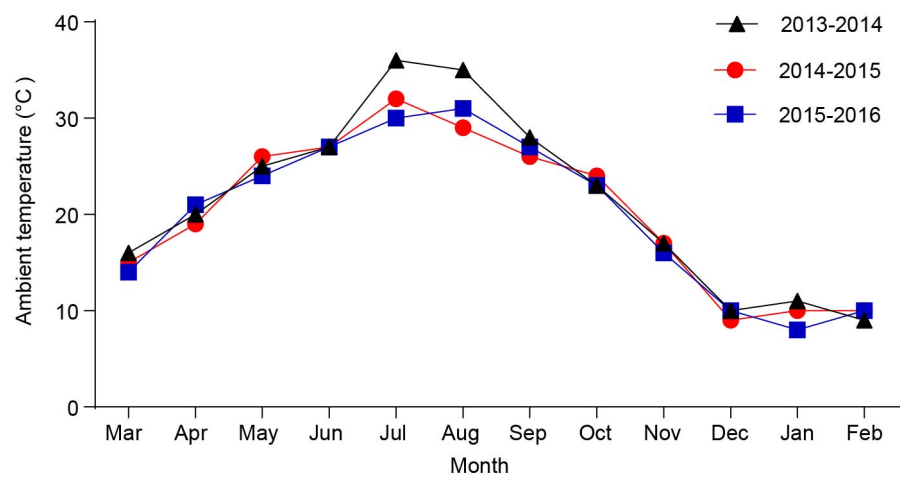

Supplement: Supplementary file 2 — Supplementary figures [file 41419_2019_1706_MOESM2_ESM.pdf]
